# Treating extravasation injuries in infants and young children: a scoping review and survey of UK NHS practice

**DOI:** 10.1186/s12887-018-1387-1

**Published:** 2019-01-07

**Authors:** Mark Corbett, David Marshall, Melissa Harden, Sam Oddie, Robert Phillips, William McGuire

**Affiliations:** University of York, York, YO10 5DD England

**Keywords:** Extravasation injury, Infants, Neonates, Children, Scoping review, Survey

## Abstract

**Background:**

Extravasation injuries are caused by unintended leakages of fluids or medicines from intravenous lines but there is no consensus on the best treatment approaches, particularly in infants and young children.

**Methods:**

This paper presents a more succinct account of a study of treatments for extravasation injuries in infants and children which has also been reported in full as an NIHR HTA report. A systematic scoping review and survey of UK NHS practice were undertaken. Twelve databases - including MEDLINE and EMBASE - were searched for relevant studies in February 2017. Studies of children with extravasation injuries receiving any treatment for extravasation injury were eligible, providing they reported one of the following outcomes: wound healing time, infection, pain, scarring, functional impairment, and requirement for surgery. Studies were screened in duplicate. Data were extracted by one researcher and checked by another. Studies were summarised narratively. An online questionnaire was distributed to NHS staff at neonatal units, paediatric intensive care units and principal oncology/haematology units.

**Results:**

The evidence identified in the scoping review was mostly comprised of small, retrospective, uncontrolled group studies or case reports. The studies covered a wide range of interventions including conservative management approaches, saline flush-out techniques (with or without prior hyaluronidase), hyaluronidase without flush-out, artificial skin treatments, debridement and plastic surgery. Few studies graded injury severity and the results sections and outcomes reported in most studies were limited. There was heterogeneity across study populations in many factors.

The survey yielded 63 responses from hospital units across the UK. Results indicated that although most units had written documentation for treating extravasation injuries, only one-third of documents included a system for grading injury severity. The most frequently used interventions were elevation of the affected area and analgesics. Saline wash-out treatments, either with or without hyaluronidase, were regularly used in about half of all neonatal units. Most responders thought a randomised controlled trial might be a viable future research design.

**Conclusions:**

There is some uncertainty about which are most the promising treatments for extravasation injuries in infants and young children. Saline flush-out techniques and conservative management approaches are commonly used and may be suitable for evaluation in trials. Although conventional randomised trials may be difficult to perform a randomised registry trial may be an appropriate alternative design.

**Electronic supplementary material:**

The online version of this article (10.1186/s12887-018-1387-1) contains supplementary material, which is available to authorized users.

## Background

Intravenous (IV) access for the provision of medication and nutrition is a common, and in many cases essential, procedure when treating children and infants in hospital. Although adverse outcomes resulting from IV access are rare, the procedure is not without risk. Extravasation injuries are caused by unintended leakages of fluids or medicines from IV lines in which a fluid deviates from its planned pathway - the vein - into surrounding tissue. These injuries can cause pain, inflammation, tendon or nerve damage and predispose to local and invasive infection, ulceration and tissue necrosis. Initial treatments aim to reduce pain and prevent or minimise local tissue necrosis and associated functional and cosmetic impairment. Injuries which result in tissue necrosis seem to be more prevalent in neonates and younger infants. This is likely to be due to their immature skin, fragile veins, lack of subcutaneous tissue, limited ability to report pain, likelihood of needing longer periods of intravenous treatment, limited number of venous access sites, the small-bore of catheters and the small drug volume. This paper presents a more succinct account of a study of treatments for extravasation injuries in infants and children which has also been reported in full as an NIHR HTA report. [1]

There is some uncertainty about the incidence of extravasation injuries in children. Across different oncology populations (including adults) reports range between 0.01 and 7% for chemotherapy extravasations. [2] A study of 1409 neonates reported a severe injury rate of 2.4% with total parenteral nutrition solution being involved in most cases. [3] Extravasation injuries have been classified into four stages of increasing severity based on assessment of pain, erythema, swelling, blanching, capillary refill, and pulse volume. [4] Although these Millam guidelines may generally be useful in predicting injury prognosis, and in determining the best treatment results, they appear to have a more limited value in paediatric populations. [5, 6]

Treatment strategies are normally driven by the type and extent of the injury, the type of infusate and by the time-interval between injury identification and subsequent intervention. Although treatment options are many and varied there is no consensus on the best approach to management, with guidelines sometimes offering conflicting recommendations. [7–9] This is likely a result of the limited research evidence available, particularly in newborns and infants. Consequently, it is unsurprising that policies seem to be largely based on historical practice within hospitals or expert opinion, rather than on published guidelines. [10] This study aimed to begin the process of resolving the uncertainty surrounding which treatments may be best for treating extravasation injuries in infants and young children. This was done by undertaking both a systematic scoping review and an NHS survey of current practice and opinions.

## Methods

### Scoping review

A scoping review was undertaken to determine which treatments appear likely to be the most promising for future study. The review was based on the framework proposed in key scoping review methodology papers. [11–13] In February 2017, the following databases were searched to identify published and unpublished studies in any language: MEDLINE, British Nursing Index (BNI), Cochrane Central Register of Controlled Trials **(**CENTRAL), Cochrane Database of Systematic Reviews (CDSR), Cumulative Index to Nursing & Allied Health (CINAHL Plus), Database of Abstracts of Reviews of Effects (DARE), EMBASE, EMCARE, Proquest Dissertations & Theses: UK & Ireland, Conference Proceedings Citation Index: Science, Health Technology Assessment (HTA) Database, Maternity and Infant Care (MIC), PubMed and Science Citation Index. We also searched three clinical trial registries for ongoing studies: ClinicalTrials.gov, EU Clinical Trials Register, and the WHO International Clinical Trials Registry Platform portal. An example search strategy (for MEDLINE) is presented in Additional file 1.

Eligible studies were of children (aged < 18 years) with an extravasation injury of the skin, subcutaneous tissue or muscle tissue, associated with central or peripheral intravenous access. Any interventions or comparators were eligible. The outcomes of interest were: wound healing time, scarring, infection, pain, contractures, functional impairment, disfigurement, requirement for surgery, mortality, and anaphylactic reactions to extravasation treatments. Any study design was eligible. We included any identified reviews (which had a focus on treatments) and guidelines to provide the basis for an overview of the evidence for extravasation treatments more broadly, i.e. studies of adults, since it was possible that some of this evidence might have been more methodologically robust than the studies in children.

Two reviewers independently assessed titles and abstracts for eligibility. The full texts of potentially relevant titles and abstracts were sought and assessed independently by two reviewers, with disagreements resolved through discussion or via a third reviewer. Piloted data extraction forms for comparative studies, non-comparative studies and case reports were used to record details of study methods, population characteristics (such as age, type of infusate, and injury severity), interventions (type, number and frequency of treatments), comparators, outcome measures, and results. Any recommendations for future research which were relevant to the aims of this scoping review were also extracted. Data were extracted by one researcher and checked by another. Studies were synthesised, and summarised narratively.

### Survey

An NHS survey was undertaken to inform on which treatment approaches are currently used and to elicit opinions regarding which interventions are most worthy of future research. A systematic approach was used to develop the questionnaire content, informed mainly by initial findings from the scoping review and peer-to-peer consultation of clinicians. The questionnaire was designed and distributed using *Qualtrics* software. It was piloted among colleagues at neonatal and paediatric units in York, Bradford and Leeds and distributed to NHS staff at neonatal units, paediatric intensive care units (PICUs) and principal oncology/haematology units nationwide. The questionnaire is presented in the Additional file 2.

## Results

### Scoping review

**Fig. 1:**
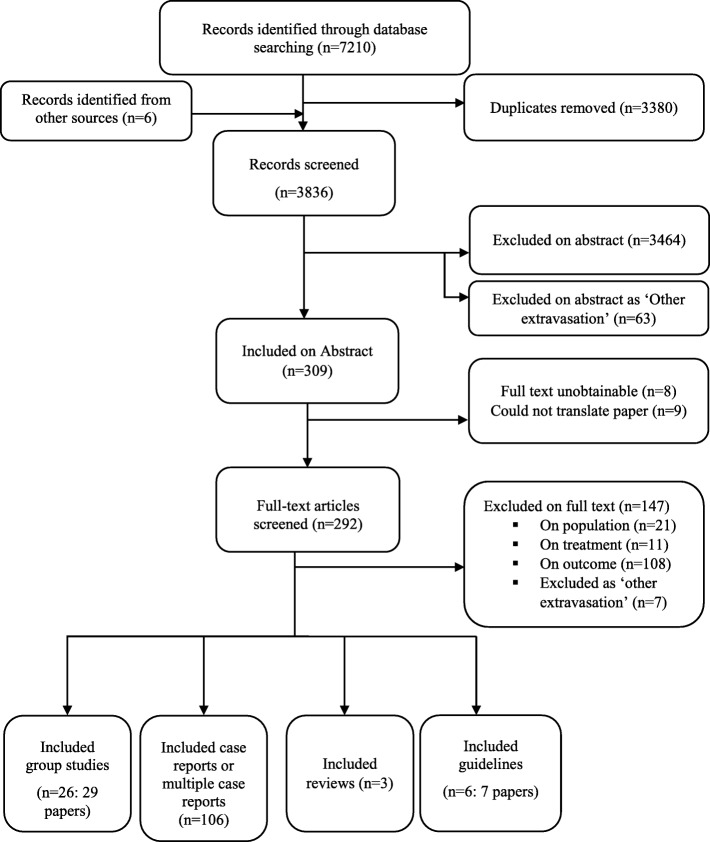
Flow chart showing the number of studies identified and eligible for scoping review inclusion

From the database searches 3830 records were identified for title and abstract screening, from which 289 records were selected as being of interest. After screening full papers we included 26 group studies, six guidelines, three reviews and 106 case report studies (Fig. 1). Case report details and references are available in Additional file 3.

Of the 26 included group studies only two were comparative (neither were randomised studies) and both had limitations. [9, 14] One was an old quasi-randomised study of treatments which are now not commonly used (silver sulfadiazine cream and povidone-iodine ointment). [14] It included 34 patients with quite severe wounds where the extravasation injuries may not have been identified for quite some time. The other study was not primarily designed to evaluate treatment effectiveness. Although it did endeavour to determine if hyaluronidase treatment resulted in less harm than no hyaluronidase treatment, the before-and-after design, lack of population details, and lack of details on interventions given to the no hyaluronidase group, mean the study’s ‘harm score’ result should not be regarded as a reliable estimate of hyaluronidase effectiveness. [9] Full details and results of both studies are in the Additional file 4.

Although many types of extravasation injury treatments have been studied in the 24 non-comparative studies which were included, the limitations inherent in these studies make it very difficult to compare results across treatments; details and results of the non-comparative studies are presented in Additional file 4. Some results are likely to have been subject to chance effects or biases because most studies were very small and were retrospective in design: 17 of the 24 studies had sample sizes of less than 20, and only three studies were reported as having a prospective design. Furthermore, there was considerable clinical heterogeneity across study populations in factors such as age, types of infusate, injury severity, location of injury, and time between injury and treatment. Differences in results across studies might be a reflection of variation in one or more of these parameters, rather than differences in treatment effectiveness. Although data on injury severity grading could have helped with interpreting the importance of these issues, few studies reported such data (only 3 of the group studies). The results sections for most studies were very brief and reported limited results data. Moreover, the reported outcomes often related to short-term time points. No studies reported pain as an outcome and few studies quantified outcomes – e.g. using measures of scarring, such as scar scores. Only one study reported on whether or not interventions resulted in adverse effects. [15]

Some of the better evidence (in terms of study size and use of a prospective design) related to studies of saline flush-out techniques, which appear to be quite promising treatments. The effect of prior infiltration with hyaluronidase before wash-out is unclear though.

Neonates were the most frequently studied population, being evaluated in around half the non-comparative studies. Sung & Lee suggested that the use of flush-out methods in neonates may be too invasive to perform and therefore proposed a middle ground between conservative management and flush-out: puncture points and hydrocolloid dressing. [16] However, although two of the 12 (mostly) pre-term neonates in this South Korean study presented with necrotic lesions, nine eventually progressed to full thickness open wounds. Besides, two group studies *have* reported results for flush-out treatments used in neonates. [3, 17] One of them was prospectively performed in a UK neonatal unit, but was published only as a letter and so only described the population as ‘neonates’ along with very basic result details. [17] The other study was conducted in a Greek neonatal intensive care unit in mostly very pre-term or late pre-term neonates with quite severe (stage III or IV) extravasation injuries. [3] This study reported impressive results with 21 of 34 neonates showing no signs of soft tissue damage 24 h after treatment, and only minor findings - blistering and epidermolysis - still present in seven neonates in the following few days. These results might therefore suggest that flush-out may be more worthy of further study than the middle ground of puncture (*without* flush-out) and dressing. However, this is merely a suggestion, as although both the studies were of parenteral nutrition extravasations in neonates they differed in an important way: in the Greek study the neonates were treated within 10–30 min of injury compared with between 1 and 10 h in the South Korean study.

All three of the identified reviews concurred that although immediate treatment is needed for the best outcomes, there is no consensus regarding which treatments constitute best practice. [7, 18, 19] They all mentioned saline washout with or without hyaluronidase as a frequently studied treatment but no review could make conclusive statements on its effectiveness compared to other treatments due to the limited quality of evidence. Seven published guidelines were identified. [2, 8, 20–24] Only one focussed specifically on a paediatric population. [22] Their recommendations were often conflicting on treatments, including saline washout, [2, 23] specific antidotes [2, 22] and conservative management. For example, the saline flush-out treatment, as originally proposed by Gault [25] has been described as very effective and to be recommended, [7] as having achieved good results, [23] as potentially effective but lacking in evidence [8] and as not to be recommended as routine management. [2] They did report similar findings on hyaluronidase (as being effective) and corticosteroids (as being ineffective).

### Survey

Sixty-three questionnaires were received from 56 different hospitals; 71% were from neonatal units, 21% were from principal oncology/haematology units and 8% were from PICUs. Forty-eight (76%) questionnaires were received from units in England, six (10%) from Scotland, five (8%) from Northern Ireland and two (3%) from Wales; two (3%) responses were received from units in North America. Most responders were either consultant neonatologists (48%), nursing staff (16%) or consultant paediatricians (13%). Of 57 responding units, 82% said they had a written protocol or guideline for treating extravasation injuries, although a staging system for grading injury severity was included in only around a third of protocols or guidelines. Almost all responders indicated that peripheral lines were the access site most associated with extravasation injuries. In neonatal units parenteral nutrition was the cause of the largest proportion of extravasation injuries. In principal oncology/haematology units the largest proportion of injuries was due to vesicant chemotherapies.

The most frequently used intervention approaches were elevation of the affected area and analgesics (see Additional file 5). In most units warm or cold compresses were rarely or never used. In neonatal units there was notable variation regarding the use of occlusive dressings, ranging from always being used (8% of responses) to never being used (31%). Variation in the use of saline flush-out, either with or without hyaluronidase, was also evident; these interventions seem to be either usually or sometimes used in around half of neonatal units, though never used in around a third of units. Results for principal oncology/haematology units and PICUs were broadly similar to the neonatal unit results.

When asked about a future research study, 65% of the 57 responders thought a randomised controlled trial (RCT) might be viable, 21% did not think an RCT was viable and 14% did not know. However, the results varied by setting: the proportion thinking an RCT was viable was 83% of the 40 neonatal unit responses, 33% of the 12 principal oncology/haematology unit responses and 0% of PICUs. Almost all the responders who thought a RCT was viable mentioned one or more of the following types of treatment when asked which treatments they would most like to see studied: saline irrigation/wash-out, hyaluronidase and conservative management. Of those who thought an RCT was not viable various reasons were provided including the presence of too many variables which could affect outcomes, timeliness of treatment when using randomisation, low numbers of patients, and unwillingness to deviate from current practice. Tables of survey results are presented in the Additional file 5.

## Discussion

Our systematic scoping review identified studies which, together, covered a wide range of treatments for extravasation injuries. However, in considering the study methods and designs used, small sample sizes, and the variation across population and intervention characteristics, the quality of evidence overall was very low. Consequently, there is uncertainty about which treatments are most promising. Notwithstanding the evidence limitations, the results of studies of flush-out techniques suggested that these treatments may be worthy of further research. This finding was echoed in the NHS survey results, with flush-out techniques, hyaluronidase and conservative management approaches frequently suggested as being treatments where further study would seem most worthwhile. Our survey results were similar to those from previous surveys - conducted in the USA [26] Australia and New Zealand [27] and Britain [28] - in demonstrating a lack of consensus on the best course of treatment for extravasation injuries. The main limitation of our study related to the scoping review evidence identified - most studies were very limited in helping to evaluate relative treatment efficacy.

In planning a future comparative study of extravasation injury treatments, population heterogeneity, low injury rates and sporadic incidence are key issues. In light of this, the most viable population for any randomised trial may be preterm neonates receiving IV parenteral nutrition at a peripheral site, although this treatment approach is quite rare, and is not usually recommended. A paucity of standardised relevant outcome measures used in previous studies in neonates is also a concern. Outcome measures used in a future study would ideally need to be clinically practicable but also be able to demonstrate adequate reliability and validity. Although a conventional parallel-group randomised controlled trial might seem the ideal design to use in a future study, it is likely to be difficult to overcome the following issues: avoiding treatment delays, selection bias and the recruitment of adequate numbers of participants. Since extravasation injuries require urgent treatment any delay due to recruitment and randomisation processes might be difficult to justify. A frequently used method of randomisation is sequentially numbered, opaque, sealed envelopes containing randomly generated treatment allocations. Adoption of this method might minimise treatment delays, but this approach has been demonstrated to be prone to investigator selection bias. [29] Extravasation injuries are quite rare events which are also subject to variation, particularly in terms of patients (ages, comorbidities), causes (infusates), injury sites and severities, and the speed at which injuries are detected and treated. Consequently, careful consideration would be needed when devising trial eligibility criteria to enable the recruitment of both a sufficiently homogeneous sample of participants and a sample which would be large enough to minimise the impact of chance differences across treatment groups in any of these factors. Failure to do so would increase the risk of false-positive trial results; small trials are more prone to yielding chance results than larger trials.

Alternatives to conventional RCT designs should therefore be considered. Although a prospective, observational database study would maximise the number of patients recruited, and eliminate concerns about treatment delays, its results would inherently be subject to uncertainty due to the likelihood of selection bias. Nevertheless, a randomised registry trial design could be used which incorporates many of the best aspects of both conventional RCTs and observational database studies. [30] Issues (highlighted by our survey results) which should be considered in any randomised registry trial of neonates include the lack of a protocol or guideline for treating extravasation injuries in some units, and the absence of the use of a system for grading injury severity in many units which *do* have access to a protocol or guideline. There may also be variation in the injury severity grading systems used; it has been argued that the Millam guidelines are not appropriate in paediatric populations [5, 6] with alternatives proposing the inclusion of assessment of the number of joints involved [5] or the percentage of the limb affected. [6]

## Conclusions

Studies of treatments for extravasation injuries in babies and children are mostly very small, lack comparator groups, and are varied in terms of patient, intervention and outcome characteristics. Consequently, there is uncertainty about which treatments are most promising. However, the results of studies of flush-out techniques suggest that these treatments may be worthy of further research. NHS survey results echoed this finding with hyaluronidase and conservative management frequently also suggested as being treatments where further study would be most worthwhile. Nevertheless, some of the practicalities involved in undertaking a conventional randomised controlled trial - such as recruiting adequate numbers, avoiding treatment delays and selection bias - could be difficult to overcome. An alternative design is the randomised registry trial, which incorporates many of the best aspects of both conventional RCTs and observational database studies.

## Additional Files

Additional file 1: Search strategy for MEDLINE (DOCX 13 kb)

Additional file 2: Survey questionnaire content and logic (DOCX 17 kb)

Additional file 3: Case report study details (DOCX 152 kb)

Additional file 4: Comparative study and non-comparative group study details (DOCX 75 kb)

Additional file 5: Survey result details (DOCX 43 kb)

## Abbreviations

CRD: Centre for Reviews and Dissemination
IV: intravenous
NHS: National Health Service
PICU: Paediatric intensive care unit
RCT: Randomised controlled trial
