# Supplementary material for: Treating extravasation injuries in infants and young children: a scoping review and survey of UK NHS practice
Source: BMC Pediatr. 2019 Jan 7;19:6. doi: 10.1186/s12887-018-1387-1 (PMC6323695; doi:10.1186/s12887-018-1387-1)
Supplement: Supplementary file 1 — Search strategy for MEDLINE (DOCX 13 kb) [file 12887_2018_1387_MOESM1_ESM.docx]

Additional file 1 - Search strategy for MEDLINE (Epub Ahead of Print, In-Process & Other Non-Indexed Citations, Ovid MEDLINE(R) Daily and Ovid MEDLINE(R))

via Ovid <http://ovidsp.ovid.com/> 1946 to present

Searched on: 1^st^ February 2017

Records retrieved: 1969

1 "Extravasation of Diagnostic and Therapeutic Materials"/ (3147)

2 extravasat$.ti,ab. (15523)

3 (infiltrat$ adj2 (intravenous$ or IV or infus$ or catheter$ or cannula$)).ti,ab. (239)

4 (infiltrat$ adj2 (injur$ or wound$)).ti,ab. (874)

5 ((intravenous$ or IV or infus$) adj2 leak$).ti,ab. (152)

6 (infus$ adj2 (injur$ or wound$)).ti,ab. (289)

7 (PIV adj2 (injur$ or wound$)).ti,ab. (2)

8 (PIV adj2 infiltrat$).ti,ab. (3)

9 (catheter$ adj2 (injur$ or wound$)).ti,ab. (708)

10 or/1-9 (19211)

11 exp Child/ (1703797)

12 exp Infant/ (1029438)

13 Adolescent/ (1782430)

14 (child$ or infant$ or infancy or pediat$ or paediat$ or preschool$ or pre school$ or schoolchild$ or school age$ or schoolage$ or schoolboy$ or schoolgirl$).ti,ab. (1552190)

15 (girl or girls or boy or boys or kid or kids).ti,ab. (201718)

16 (adoles$ or young people or young person$ or teen$ or youth$ or preteen$ or juvenil$).ti,ab. (345875)

17 (neonat$ or neo nat$).ti,ab. (225315)

18 (newborn$ or new born$ or newly born$).ti,ab. (146891)

19 (preterm or preterms or pre term or pre terms).ti,ab. (58825)

20 (preemie$ or premie or premies).ti,ab. (140)

21 (prematur$ adj3 (birth$ or born or deliver$)).ti,ab. (13561)

22 (low adj3 (birthweight$ or birth weight$)).ti,ab. (29760)

23 (lbw or vlbw or elbw).ti,ab. (6880)

24 (baby or babies).ti,ab. (60639)

25 or/11-24 (3777516)

26 10 and 25 (2131)

27 exp animals/ not humans/ (4311358)

28 26 not 27 (1969)

**Key:**

/ = indexing term (MeSH heading)

exp = exploded indexing term (MeSH heading)

$ = truncation

ti,ab = terms in either title or abstract fields

adj2 = terms within two words of each other (any order)
