# Supplementary material for: Treating extravasation injuries in infants and young children: a scoping review and survey of UK NHS practice
Source: BMC Pediatr. 2019 Jan 7;19:6. doi: 10.1186/s12887-018-1387-1 (PMC6323695; doi:10.1186/s12887-018-1387-1)
Supplement: Supplementary file 2 — Survey questionnaire content and logic (DOCX 17 kb) [file 12887_2018_1387_MOESM2_ESM.docx]

Additional file 2 - Questionnaire content and logic

| **Question** | **Answers and format** |
| --- | --- |
| What is your position? (e.g. consultant paediatrician): | Free text |
| What is the name and location of your unit? | Free text |
| Which of the following best describes your clinical setting: | - Neonatal Unit - Paediatric Intensive Care Unit - Principle Oncology/Haematology unit - Shared Care Oncology/Haematology unit - Other (please state) |
| Does your unit have a written protocol or guideline for treating extravasation injuries?  If yes, ask:   - Does the protocol or guideline contain a staging system for grading severity of extravasation injury? | - Yes - No - Yes - No |
| Does your unit have a list of treatments/interventions which may cause serious problems when extravasated? | - Yes - No |
| Please consider the list below of possible treatments for extravasation injuries. How frequently is each of them used in your unit?   - Elevation of affected area - Warm compress - Cold compress - Analgesia - Specific topical cream or ointment (please state) - Occlusive dressing - Saline irrigation ***without*** hyaluronidase - Saline irrigation ***with*** hyaluronidase - Antidotes to specific infusates | For each, choose one of:   - Always - Usually - Sometimes - Rarely - Never - Don’t know |
| Apart from plastic surgery, are there any other interventions you would use for extravasation injuries which were not listed in the previous question?  If Yes please list the other intervention(s) and indicate frequency of use. **Please note that our study does not cover preventative interventions.** | - Yes - No   Free text along with one of:   - Always - Usually - Sometimes - Rarely |
| Please select the type of access site most associated with extravastion injuries in your unit’s patients: | - Peripheral line (hands, feet) - Peripheral central line - Central line - Other (please state) - Don’t know |
| Please select the type of infusate which causes the largest proportion of all the extravastion injuries in your unit’s patients. | More than one option maybe selected if the proportion of injuries is around the same for 2 or more types of infusate:   - Parenteral nutrition - Contrast agents - Calcium - Blood - Vesicant chemotherapies - Non-vesicant chemotherapies - Inotropes or pressors - Other (please state) - Don’t know |
| What proportion of the extravasation injuries in your unit would you estimate is caused by extravasation of infused [insert above response] | - 75-100% - 50-74% - 25-49% - 11-24% - 1-10% - Not sure, but more than 50% - Not sure, but less than 50% |
| Approximately what proportion of extravasations injuries that you have actively treated have resulted in a need for plastic surgery at any stage? | - More than 50% - 25-50% - 5-24% - Less than 5% - Don’t know |
| In the last 10 years did any of the extravasation injuries which occured in your unit result in litigation?   - If Yes, please state how many cases resulted in litigation: | - Yes - No - 1 - 2 - 3 - 4 - 5 - 6 or more - Don’t know |
| Regarding a future research study in this area, do you think a randomised trial design can be successfully undertaken to compare different treatments for extravasation injuries in babies and young children?  If yes:   - Please tell us which treatment(s) you would most like to see studied in a randomised trial (state one or two treatments):   If no:   - It would be helpful if you could say why a randomised trial design might not be viable. If you have any thoughts on alternative study designs, which you think might be more appropriate, please also state them here. | - Yes - No   Free text  Free text |
| Are you aware of any summary data on the effectiveness or safety of treatments for extravasation injury which we are unlikely to have identified in our searches of literature databases (e.g. unpublished data)?   - If Y display “We would be very grateful if you could email details on the summary data to mark.corbett@york.ac.uk within the next three weeks.” | - Yes - No |
| If you have any comments or suggestions about our study which haven't been covered in this survey please add them here | Free text |
| Would you like to receive an email notification when our final report is published online (it will be open-access)?   - If Y display “Please tell us the email address you would like the link sending to:” | - Yes - No thanks   Free text |
| End message: Thank you very much for completing the survey. |  |
