# Supplementary material for: Treating extravasation injuries in infants and young children: a scoping review and survey of UK NHS practice
Source: BMC Pediatr. 2019 Jan 7;19:6. doi: 10.1186/s12887-018-1387-1 (PMC6323695; doi:10.1186/s12887-018-1387-1)
Supplement: Supplementary file 3 — Case report study details (DOCX 152 kb) [file 12887_2018_1387_MOESM3_ESM.docx]

Additional file 3 Case report details

| **Authors** | **Design** | **Age** | **Infusate** | **Intervention** | **Outcome** |
| --- | --- | --- | --- | --- | --- |
| Abraham et al. 2012[1] | **Case report** | **9 years** | **Arginine and 10% glucose** | **Cool compresses and dressings** | **Residual scar but no other complications** |
| Altan et al. 2013[2] | **Case report** | **23 days** | **Contrast agent** | **Elevation and cold compresses. Volar fasciotomy for compartmental syndrome** | **No functional complications** |
| Altmann et al. 2014[3] | **Extractable: Only 1 case (rest mixed with adult population).** | **2 years** | **Unspecified antibiotic** | 1. **Radical debridement** 2. **Wound conditioning EITHER by vacuum assisted closure (V.A.C.) OR t**emporary wound coverage by allogeneic donor-tissue grafts (unclear which) 3. **Full-thickness skin-graft** | **Full restoration of right-hand function** |
| Amano et al. 2008[4] | **Case report** | **3 years** | **Arginine monohydrochloride (10% in NaCl)** | **Conservative therapy with 1% silver sulphadiazine** | **One month after the accident the ulcer healed, leaving a slight hypertrophic scar.** |
| Amaya 2016[5] | Multiple case reports (4 patients) | **4-32 weeks old (3 preterm)** | **NR** | **Active leptospermum honey, debridement and dehydrated amniotic membrane allograft** | **Needed skin graft but no untoward effects seen (healed 21 to 41 days).** |
| Amhaz et al. 2016[6] | **Case report** | **10 days** | **Blood** | **Lipoaspiration cannula to evacuate the hematoma, elevation and compress** | **Healed over 2 weeks** |
| Aribit et al. 2000[7] | **Multiple case reports (2 patients)** | **6 and 11 months** | **Glucose 10% (1), NR (1)** | **Lipoaspiration,** Followed by local care until healed. | **Full recovery at 4 months other than post-epidermolysis dyschromia, no other trophic or neurological adverse event** |
| Baker et al. 1991[8] | **Case report** | **7 years** | **Arginine monohydrochloride (10%)** | **Elevate, cold compress. Topical silver sulphadiazine, after twice daily hydrodebridement. Surgical debridement, and skin graft.** | **Nerve and tendons destroyed. Skin graft necessary and was 98% viable fully functional after 5 days. Full function at 4 weeks.** |
| Bassi et al. 2007[9] | **Case report** | **10 months** | 6 cc arginine monohydrochloride, 50% diluted in 12 cc of sodium chloride 0.9% | **Managed conservatively. Enzymatic debridement by collagenase ointment (clostridiopeptidase A) together with local antiseptics** | **Complete resolution within 2 months with hypertrophic scar (Fig. 2). There was no need for skin grafting.** |
| Berger et al. 1974[10] | Multiple case reports (3 patients) | **2 days to one month (2 preterm)** | **All calcium gluconate** | **Soaks and mechanical debridement in 2. Antibiotics in 2** | **Took between 3 weeks and 6 months to heal depending on severity** |
| Beytut et al. 2014[11] | **Case report** | **7 years** | **NR** | **Oxygenotherapy, heat treatment and dressings with dextrose** | **12 days to full recovery** |
| Bhosale et al. 2012[12] | **Case report** | **16 years** | **Dopamine** | 4 days of antibiotics, and noradrenaline with dopamine. Followed by skin debridement and grafting. | **Needed skin graft.** |
| Borman et al. 1998[13] | **Case report** | **4 years** | **Chloramphenicol and ampicillin** | **Dermatofasciotomy, heparin infusion** | **Day 20 gangrene and amputation of hand** |
| Boyar et al. 2014[14] | **Case report** | **3 weeks (preterm)** | **NR** | **Medihoney gel and dressing** | **Healed over 3 weeks with some scarring** |
| Broom et al. 2016[15] | Multiple case reports (2 patients) | **6 months to 1 year** | **NR** | **All underwent fasciotomy for compartment syndrome** | **Both had excellent outcome** |
| Chait et al. 1975[16] | **Case report** | **2 years** | **Oncovin dauno rubicin** | **Moist dressings and elevation** | **Healed within 3 months with some scarring** |
| Chen et al. 2010[17] | **Case report** | **4 days (preterm)** | **Calcium gluconate (10%)** | **Elevation, cold packs. Oxacillin, ampicillin and gentamicin, fasciotomy (x2) for compartment syndrome, vancomycin and ceftazidime. Wet dressings.** | **Improved after 3 months** |
| Chiang et al. 2004[18] | **Case report** | **11 days (preterm)** | **Calcium gluconate (10%)** | **Elevation, cold packs, oxacillin and gentamicin, vancomycin (4 weeks)** | **Improved after 45 days** |
| Ching et al. 2014[19] | **Case report** | **4 days** | Calcium gluconate | **Managed conservatively** | **Improved after 20 weeks** |
| Cho et al. 2007[20] | Multiple case reports (5 patients) | **17 to 50 days** | **Parenteral nutrition**  **(6^th^ case blood transfusion)** | **Antibacterial (antibiotic) ointment, sesame oil, anti-inflammatory herbal mixture, dressings (1 debridement, 1 escharectomy and oral antibiotic), Vitamin C.** | **1 mth to 2 yrs: no scar and no functional abnormalities** |
| Cohan et al. 1990[21] | Case report | **12 months** | **Iopamidol** | **Elevation and warm compresses** | **2 days to full recovery** |
| D’Acunto et al. 2015[22] | Case report | **2 months (preterm)** | **Balanced electrolyte solution** | **Elevation, proteolytic cream, escharectomy as well as autograft skin** | **Total recovery after 1 year** |
| Dave 1993[23] | Case report | **3 years** | **Undefined fluids (no drugs)** | **Wet gauze, hot packs, debridement, and skin graft** | **No long-term functional complications** |
| Davies et al. 1994[24] | Multiple case reports (2 patients) | **26 and 11 days (both preterm)** | Parenteral nutrition | **Subcutaneous hyaluronidase and saline flushing** | **Healed with minimal scarring/no sign of injury** |
| Denkler et al. 1989[25] | Case report | **1 day (preterm – two sites, hand/foot)** | **Dopamine** | **2% nitroglycerin ointment and elevation** | **Full recovery same day** |
| Domizio et al. 2006[26] | Case report | **2 days (2 sites)** | **Ampicillin (50 mg/kg/day) and Cefotaxime (100 mg/kg/die) added with 10% calcium gluconate** | **7 days later treated topically with an antibiotic-corticosteroid cream** | **20 days later, only whitish subcutaneous nodules from which tiny white pieces of calcific masses were eliminated spontaneously without signs of inflammation** |
| Dunn et al. 1984[27] | Case report | **5 months** | **Dextrose and 25% normal saline** | **Elevation** | **2 months later, no movement or feeling in parts of hand. 9 months after injury, improvement** |
| Duray et al. 1986[28] | Case report | **5 years** | **Doxorubicin** | **Excision of surrounding skin** | **Skin graft needed but healed** |
| Eckersall et al. 1996[29] | Case report | **3 years** | **Dextrose saline** | **Elevation (24 hours)** | **3 days to full recovery** |
| Eroglu et al. 2004[30] | Case report | **17 years** | **Mannitol (20%)** | **Fasciotomy for compartment syndrome** | **Fully functional, with a scar** |
| Garcia-Alverez et al. 1999[31] | **Case report** | **2 weeks (administered over first 3 days of life)** | **Calcium gluconate** | **Managed conservatively** | **Full recovery 10 weeks later** |
| Gibboney et al. 1986[32] | Multiple case reports (2 patients) | **17 days and 4 weeks (both preterm)** | **IV fluids** | **Surgical debridement and antibiotics, one received several skin grafts** | **5.5 to 9 months, healed** |
| Govind et al. 2014[33] | Case report | **27 days (preterm)** | **Parenteral nutrition (lipid infusate)** | **Incision and drainage, flushing of central line** | **18 months, healed naturally** |
| Grabois et al. 2008[34] | Case report | **19 days (preterm)** | **Sodium bicarbonate** | **Clean wound and covered with Vaseline (sterile petrolatum) for 20 days** | **Recovered after 20 days** |
| Handler 1990[35] | Case report | **4 years** | **Dextrose solution (5%), 25% saline, and KCl** | **Elevated and warm dressing.** Fasciotomy for compartment syndrome, skin graft | **Needed skin graft** |
| Hankin et al. 1984[36] | Case report | **17 years** | **Doxorubicin** | **Cold packs. Wet to dry dressings. Conservative management. Debridement after 7 months** | **Healed with contracture of arm** |
| Harb et al. 2010[37] | Case report | **1 year (preterm)** | **Erythromycin** | **Area was irrigated with saline through small punctures in the skin around the injury site. Managed initially conservatively, with regular dressing changes and delayed surgical intervention. At 3 weeks – debridement and skin graft** | **One week later – healing well** |
| Hasija et al. 2014[38] | Case report | **3 years** | **Phenytoin** | **Fasciotomy for compartment syndrome** | **After a few days, normal tissue texture and the injury was managed** |
| Hey et al. 2005[39] | Case report | **12 months** | **Azithomycin** | **Warm compresses, adaptic dressing, splint, elevation, topical antibiotics** | **Small area of unusual pigmentation, but otherwise healthy** |
| Hironaja et al. 1982[40] | Case report | **6 days** | **Calcium gluconate (10%)** | **Warm soaks, Debridement, soaks of (2%) ethylenediaminetetraacetic acid** | **At 4 months, full recovery but does not mention scarring etc.** |
| Hirsch et al. 2016[41] | Case report | **4 days (preterm)** | **Parenteral nutrition** | **Elevated, antibiotic ointment, slightly compressive gauze, 13 days debridement, collagenase, silicone dressing, splint, silver nitrite, Apligraf** | **Day 16 wound closure, At 2 years, wrist contracture** |
| Hooke 2005[42] | Case report | **Adolescent** | **Doxorubicin** | **Aspirated, cold packs dimethyl sulphoxide (DMSO) solutions topically, 3 debridements, skin graft** | **Large scar but full use of area** |
| Kameo et al. 2015[43] | Case report | **2 years** | **Vincristine** | **Hyaluronidase for 3 days, warm compresses** | **Full recovery** |
| Khan et al. 2014[44] | Case report | **29 days (preterm)** | **Parenteral nutrition** | **Hyaluronidase and** bacitracin | **After 9 days , full recovery** |
| Kishi et al. 2014[45] | Case report | **17 years** | **Hydroxyzine** | **Conservative therapy and 1% silver sulphadiazine** | **After 2.5 months, slight scarring** |
| Kuensting 2010[46] | Case report | **6 days** | **10% dextrose and 0.25% normal saline solution administered at 10mL per hour with the addition of ampicillin (135 mg every 8 hours) and cefotaxime (135mg every 8 hours)** | **Elevation and warm packs, hyaluronidase, general wound care** | **Recovered within 24 hours, discharged at 8 days.** |
| Kumar et al. 2001[47] | Multiple case reports (6 patients) | **Neonate (preterm) to 2 years** | **Flucloxacillin, calcium gluconate, human immunoglobulin, sodium bicarbonate, dextrose solution, 20% lipid nutrition** | **Dressings for 3. split skin graft and debridement for 3, elevation, warm packs.** | **2 excellent (1 with scar), 1 fair, 3 moderate scarring (1 contractures treated)** |
| Lee et al. 2013[48] | Case report | **1 month (preterm)** | **Sodium bicarbonate** | **Hyaluronidase, epithelial growth factor dressings, platelet-rich plasma dressings** | **4 days after PRP, completely healed with no limitation of movement** |
| Lehr et al. 2004[49] | Multiple case reports (3 patients) | **4 to 24 days (2 preterm)** | Parenteral nutrition (lipids) plus antibiotics | **Compression, elevation, hydroactive gel** | **Healed no complications** |
| Leung et al. 1980[50] | Case report | **6.5 years** | **Contrast medium (sodium iothalamate 54%)** | **Repeated incisions, antibiotics, excision of necrotic skin, skin grafts** | **At six months, scarring, no limits on movement** |
| Llinares et al. 2005[51] | Case report | **4 years** | **Anthracycline (idarubicin)** | **Topical DMSO and cooling, antiseptic, moisturiser** | **Pain from application but recovered. At 4 weeks, loss pigmentation and focal induration** |
| Martin et al. 1994[52] | Case report | **4 months** | 8.4% bicarbonate 20 ml, 10% calcium gluconate 10 ml, 50 % glucose 5 ml, 1:1000 adrenaline 3 ml and 4.5 % human albumin solution 50 ml. | **Hyaluronidase, liposuction and saline washout** | **2 weeks later, no signs of soft tissue damage** |
| Meszes et al. 2017[53] | Multiple case reports (6 patients) | **Neonates (1 to 23 days)** | **Fatty acid, lipid and amino acid infusion (4), glucose (1), dobutamine (1)** | **Epithelising ointment (3), hydrogels (2),**  **surgical necrectomy (1), observation (1)** | **Transfer to NICU (4), home (1), surgery (1)** |
| Mohr et al. 2014[54] | Multiple case reports (2 patients) | **3 weeks (preterm), 19 days (preterm)** | **Antibiotics, NR** | **Hyaluronidase, active leptospermum honey (ALH), hydrogel, ALH calcium alginate, silver/collagen dressings** | **No negative side effects** |
| Morrison et al. 1999[55] | Multiple case reports (4 patients) | **Neonates (preterm)** | **Calcium gluconate** | **Skin grafts** | **3 years later, visible scarring** |
| Mukherjee et al. 1977[56] | Multiple case reports (2 patients) | **5 years, and NR** | **Dextrose solution; NR (rehydration)** | **Skin grafts; debridement** | **Gangrene, disfigured, and incapacitated** |
| Nissim et al. 2008[57] | Case report | **1 day** | **NR** | **Conservative treatment** | **Interval shrinkage and dissolution of the mass** |
| Onesti et al. 2012[58] | Case report | **2 days (preterm)** | **Parenteral nutrition** | **Elevation, topical silver sulphadiazine, some debridement, acellular dermal substitute, autologous keratinocytes** | **9 months, scars and deformed foot (surgical correction); 14 months, fully healed** |
| O’Reilly et al. 1988[59] | Case report | **Neonate** | **Parenteral nutrition** | **Glyceryl trinitrate patch** | **Healed without scarring (small area not covered, skin lost)** |
| Ozcan et al. 2015[60] | Case report | **14 years** | **Adrenalin** | **Elevation, local antibiotic ointment and pentoxyphilline** | **Patient died due to septic shock** |
| Pantelides et al. 2013[61] | Case report | **1 day (preterm)** | **Dextrose solution (12.5%)** | **Elevation** | **No scarring or functional deficit** |
| Park et al. 2015[62] | Case report | **7 months** | **Parenteral nutrition** | **Fasciotomy for compartment syndrome. Irrigation with saline solution. Debridement, after 4 months skin graft** | **Needed rehabilitation for contracture, healed with scar** |
| Phillips et al. 2009[63] | Case report | **3 months** | **Dopamine** | **Conservative treatment, topical antibiotics, debridement, physical therapy** | **Needed 12 months of physical therapy. May need secondary surgery** |
| Raffaella et al. 2009[64] | Case report (2 extravasations) | **5 years** | **Calcium gluconate** | **Treated conservatively (limb elevation, daily wound care, and warm compresses), antibiotics, disinfection, and physiotherapy, daily hyperbaric oxygen therapy (HOT), weekly surgical debridement and escharectomy, sodium thiosulphate for calcification** | **8 months to fully healed** |
| Ravenel 1983[65] | Case report | **6 days** | **Calcium gluconate** | **Antibiotics, nafcillin sodium** | **6 weeks, swelling subsided** |
| Reilly et al. 1977[66] | Multiple case reports (3 patients) | **13, 15 and 17 years** | **Adriamycin** | **Cold compress (1), antibiotics (1), hydrocortisone (1)** | **2 lost functional use, less serious 1 healed** |
| Reynolds 2007[67] | Case report | **2 days (preterm)** | **Intralipid and parenteral nutrition** | **Elevation** | **12 hours to heal completely** |
| Roberts 1977[68] | Multiple case reports (5 patients) | **Neonates (1 day to 1 year)** | **Calcium gluconate** | **Conservative treatment (none or warm soaks)** | **Resolved spontaneously** |
| Rosales et al. 2004[69] | Case report | **75 days (preterm)** | **Parenteral nutrition and intralipid** | **Antibiotics, drained** | **Died of sepsis** |
| Roth et al. 2006[70] | Case report | **31 days** | **Propofol and lidocaine** | **Saline, debridement, skin graft** | **Satisfactory functional healing** |
| Rustogi et al. 2005[71] | Case report | **4 days (preterm)** | **Sodium bicarbonate (NaHCO_3_)** | **Acticoat dressing** | **57 days to heal** |
| Salameh et al. 2004[72] | Case report | **3.5 years** | **Arginine** | **Compressive dressing, debridement, skin grafts** | **Functional result** |
| Samiee-Zafarghandy et al. 2014[73] | Case report | **1 day (preterm)** | **Packed red blood cells** | **Conservative management, topical nitroglycerin** | **Loss of two toes** |
| Sanpera et al. 1994[74] | Multiple case reports (2 patients) | **3 days and neonate (preterm)** | **Calcium solution and NR** | **Eusol Solution and debridements. dressings** | **Limb shortening and deformity** |
| Santoshi et al. 2008[75] | Case report | **Neonate (preterm) (seen at 5 years)** | **Blood, fluids and antibiotics** | **NR – claw deformity at 5 years - fibrous sheet was excised, the extensor tendons were tenolysed, and full correction was obtained** | **Some scarring but functional** |
| Schafer et al. 2005[76] | Case report | **2 weeks** | **Phenobarbital** | **Topical antibiotics, debridement and skin graft** | **Fully recovered** |
| Schie et al. 2013[77] | Case report | **33 weeks (preterm)** | **NR** | **Non-contact low-frequency ultrasound (NFLU; 19 sessions), debridement, amorphous hydrogel and covered with a thin film or hydrocolloid, silicone sheet** | **32 days, healed without complication** |
| Schumacher et al. 1987[78] | Case report | **7 years** | **Calcium disodium edetate (EDTA)** | **Warm soaks and splints** | **Calcification needed surgery (1.75 years later)** |
| Sharief et al. 1994[79] | Case report (2 extravasations) | **1 day (and 3 days)** | **Phenytoin** | **NR** | **1 week, complete resolution** |
| Shenaq et al. 1996[80] | Case report | **10 years** | **Adriamycin (doxorubicin)** | **Left for 4 months, Debridement, physical therapy, dressings, skin graft, capsulotomies** | **Not fully functional** |
| Sindal et al. 2015[81] | Case report | **Neonate (preterm)** | **NR** | **Debridement and topical antibiotic ointment** | **2 weeks, healed completely** |
| Siu et al. 2007[82] | Case report | **2 days (preterm)** | **Parenteral nutrition (dextrose, calcium, potassium, etc)** | **Hyaluronidase and saline flushes, dressings** | **Healed within 5 days** |
| Siwy et al. 1987[83] | Case report | **2 days** | **Dopamine** | **Infusion of phentolamine (Regitine) in saline solution, kept at heart level** | **Healed after 9 days** |
| Sokol et al. 1998[84] | Case report | **14 months (preterm)** | **Phenytoin** | **Hyaluronidase** | **Barely visible scar** |
| Sonohata et al. 2006[85] | Case report | **14 years** | **Phenytoin (diazepam before)** | **Hydrocortisone injections, elevation, warm packs** | **5 weeks to fully recovered** |
| Sonohata et al. 2008[86] | Case report | **3 days** | **Calcium gluconate** | **No treatment** | **5 months, fully recovered** |
| Soon et al. 2001[87] | Case report | **38 weeks** | Calcium gluconate | **Local skin care and topical antibiotic** | **3 months, recovered** |
| Spenny et al. 2004[88] | Case report | **3 years** | **Ceftriaxone sodium** | **Cold pack, diphenhydramine hydrochloride and epinephrine, clindamycin and morphine, fasciotomies** | **3 months, healed with complete function** |
| Stahl et al. 2000[89] | Case report | **10 years** | **Mannitol** | **Fasciotomies** | **1 year, no neurological or vascular damage** |
| Subedi et al. 2011[90] | Case report | **16 years** | **Dextrose** | **Analgesics and antibiotics followed by local incision and drainage. Managed conservatively for almost 5 months. Oral medications (gabapentin, amitriptyline, tramadol), a series of stellate ganglion blocks with bupivacaine, and limb physiotherapy** | **6 months, pain and swelling subsided drastically with marked functional recovery** |
| Subhani et al. 2001[91] | Case report | **1 day** | **Dopamine** | **Phentolamine** | **Within the next few hours, there was complete resolution of the discoloration** |
| Talbot et al. 2011[92] | Multiple case reports (3 patients) | **7 to 10 months** | **2 NR, 1 hydration** | **Fasciotomies for compartment syndrome, vacuum-assisted closure (2) or moist dressings (1)** | **Full functional recovery** |
| Tilden et al. 1980[93] | Multiple case reports (4 patients) | **15 days to 4 months** | **Nafcillin sodium** | **Saline dressing and sulphadiazine silver (2), debridement and skin graft (1), bacitracin ointment (1)** | **Healing well (3), , bacitacin not improved, died** |
| Tiras et al. 2005[94] | Case report | **2 days** | **Calcium gluconate** | **Debridement using collagenase clostridipeptidase A (CCA) and bacitracin ointment mixture in gauze after wetting the wound with sterile saline** | **Healed without surgery** |
| Tobin 2007[95] | Case report | **1 day (preterm)** | **Parenteral nutrition** | **Oral antibiotics, ActiFormCool dressings** | **6 weeks, the wound had healed, with scarring** |
| Tuncer et al. 2006[96] | Case report | **6 years** | **Calcium solution** | **Surgery for calcinosis (4 years later)** | **Full recovery** |
| Vanwijck and Lengele 1994 [97] | **Case Report** | **9 years** | Meglumine ioxitalamate | Lipoaspiration with saline wash, followed by liposuction, under LA (8) or GA (1). Redon’s drain kept under aspiration for 24h. perioperative and postoperative IV antibiotics, NSAIDS, elevated arm with light compress for 48h. Lymphatic drainage for persistent oedema at 1 week f-u. | Absent pulse in one child re-appeared immediately after liposuction. Moderate reduction in extension (20 degrees) of two fingers extension in one patient. No other adverse events. |
| **Von Mühlendahl 2012[98]** | **Multiple case reports (6 patients)** | **14 days (preterm) to 14 months** | **Fluids or electrolyte solution (5; 1 plus erythromycin), phenytoin (1)** | **Immediate/early stage: Within 24 hours of extravasation injury, complete removal of the aggravating substance via pressure relieving incisions and flushing with Ringer's solution or removal by aspiration (whichever is more appropriate); or**  **Later than 24 hours: Debridement and defect coverage (e.g., grafts)**  **Standard care (6), skin grafts (2)** | **Scars (3), loss of fingers due to sepsis (1), successful grafts (2)** |
| Wada et al. 2003[99] | Case report | **Neonate** | **Calcium solution** | **Conservative treatment, debridement, skin graft** | **6 years, surgery for physeal arrest and short leg; further surgeries up until 12 years; 16 years, deformity remained** |
| Wiegand et al. 2010[100] | Case report | **17 years** | **Dextrose** | **Elevation, cold compresses, hyaluronidase** | **Full recovery** |
| Wolfe et al. 1983[101] | Case report | **2 days** | **Calcium solution** | **Antibiotics, immobilisation, and dressings** | **6 months, full recovery** |
| Wong et al. 1992[102] | Multiple case reports (2 patients) | **4 and 15 days (both preterm)** | **Dopamine** | **Nitroglycerin ointment, phentolamine (1), elevation (1)** | **24 hours, full recovery** |
| Wong et al. 2015[103] | Case report | **4 days** | **Calcium gluconate** | **Managed conservatively** | **20 weeks, healed** |
| Yamamoto et al. 1994[104] | Multiple case reports (2 patients) | **1 and 4 years** | **Dopamine and tromethamine** | **Debridement and skin grafts (2), scar surgery (1)** | **Functional recovery** |
| Yosowitz et al. 1975[105] | Multiple case reports (7 patients) | **2 days to 10 years (2 preterm)** | **Dextrose (10%) or calcium solutions** | **Debridement (7) and skin grafts (4)** | **3 functional, 1 NR, 2 healed, 1 leg amputated** |
| Zenk et al. 1981[106] | Multiple case reports (3 patients) | **3 days to 4 months** | **Nafcillin sodium** | **Hyaluronidase (2); warm compresses and elevation, and 2 months skin graft (1)** | **3 healed (2 given hyaluronidase healed within a day)** |

1. Abraham MB, van der Westhuyzen J, Khanna V. Arginine extravasation leading to skin necrosis. J Paediatr Child Health 2012;48:E96-7.

2. Altan E, Tutar O, Senaran H, Aydin K, Acar MA, Yalcin L. Forearm compartment syndrome of a newborn associated with extravasation of contrast agent. Case Rep Orthop 2013;2013:638159.

3. Altmann S, Damert HG, Schneider W. [Clinical manifestation of extravasation caused by infusion and its therapeutic management]. Zentralbl Chir 2014;139:83-8.

4. Amano H, Nagai Y, Kowase T, Ishikawa O. Cutaneous necrosis induced by extravasation of arginine monohydrochloride. Acta Derm Venereol 2008;88:310-1.

5. Amaya R. Use of Active Leptospermum Honey (ALH) and Dehydrated Amniotic Membrane Allograft (DAMA) to manage extravasation wounds in neonates. J Wound Ostomy Continence Nurs 2016;43:S25.

6. Amhaz HH, Buretta K, Jooste EH, Machovec K, Marcus JR, Ames WA. Upper extremity peripheral intravenous line infiltration with concomitant loss of pulses treated with lipoaspiration: a case report. A A Case Rep 2016;7:185-7.

7. Aribit F, Laville J, Baron J. [Extravasation injuries and subcutaneous aspiration in children]. Rev Chir Orthop Reparatrice Appar Mot 2000;86:87-8.

8. Baker GL, Franklin JD. Management of arginine monohydrochloride extravasation in the forearm. South Med J 1991;84:381-4.

9. Bassi E, Lonati D, Pandolfi R, Gatti M, Jolliffe VM, Del Forno C. Case of skin necrosis due to arginine monohydrochloride extravasation. J Dermatol 2007;34:198-200.

10. Berger PE, Heidelberger KP, Poznanski AK. Extravasation of calcium gluconate as a cause of soft tissue calcification in infancy. Am J Roentgenol Radium Ther Nucl Med 1974;121:109-17.

11. Beytut D, Ozdamar N, Isler N, Turgut N. Extravasation injury: lokal oxigene therapy application within pediatric intensive care unit. Pediatr Crit Care Med 2014;1):134-5.

12. Bhosale GP, Shah VR. Extravasation injury due to dopamine infusion leading to dermal necrosis and gangrene. J Anaesthesiol Clin Pharmacol 2012;28:534-5.

13. Borman H, Tuncali D, Apak A, Kostakoglu N. Progressive gangrene of the hand following extravasation of antibiotics associated with hereditary resistance to activated protein C. Ann Plast Surg 1998;41:194-6.

14. Boyar V, Handa D, Clemens K, Shimborske D. Clinical experience with Leptospermum honey use for treatment of hard to heal neonatal wounds: case series. J Perinatol 2014;34:161-3.

15. Broom A, Schur MD, Arkader A, Flynn J, Gornitzky A, Choi PD. Compartment syndrome in infants and toddlers. J Child Orthop 2016;10:453-60.

16. Chait LA, Dinner MI. Ulceration caused by cytotoxic drugs. S Afr Med J 1975;49:1935-6.

17. Chen TK, Yang CY, Chen SJ. Calcinosis cutis complicated by compartment syndrome following extravasation of calcium gluconate in a neonate: a case report. Pediatr Neonatol 2010;51:238-41.

18. Chiang MC, Chou YH, Wang CR, Huang CC. Extravasation of calcium gluconate concomitant with osteomyelitis in a neonate. Acta Paediatr Taiwan 2004;45:35-7.

19. Ching DL, Wong KY, Milroy C. Iatrogenic calcinosis cutis following a neonatal extravasation injury. Br J Hosp Med 2014;75:295.

20. Cho KY, Lee SJ, Burm JS, Park EA. Successful combined treatment with total parenteral nutrition fluid extravasation injuries in preterm infants. J Korean Med Sci 2007;22:588-94.

21. Cohan RH, Dunnick NR, Leder RA, Baker ME. Extravasation of nonionic radiologic contrast media: efficacy of conservative treatment. Radiology 1990;176:65-7.

22. D'Acunto C, Neri I, Purpura V, Orlandi C, Melandri D. Extravasation injury of balanced electrolyte solution simulates the clinical condition of necrotizing fasciitis: a case report. J Pediatr Surg Case Rep 2015;3:466-8.

23. Dave AL. Third-degree burn following use of microwave-heated cryogel pack. Clin Pediatr 1993;32:191-2.

24. Davies J, Gault D, Buchdahl R. Preventing the scars of neonatal intensive care. Arch Dis Child Fetal Neonatal Ed 1994;70:F50-1.

25. Denkler KA, Cohen BE. Reversal of dopamine extravasation injury with topical nitroglycerin ointment. Plast Reconstr Surg 1989;84:811-3.

26. Domizio S, Puglielli C, Barbante E, Sabatino G, Amerio P, Artese O, et al. Calcinosis cutis in a newborn caused by minimal calcium gluconate extravasation. Int J Dermatol 2006;45:1439-40.

27. Dunn D, Wilensky M. Median and ulnar nerve palsies after infiltration of intravenous fluid. South Med J 1984;77:1345.

28. Duray PH, Cuono CB, Madri JA. Demonstration of cutaneous doxorubicin extravasation by rhodamine-filtered fluorescence microscopy. J Surg Oncol 1986;31:21-5.

29. Eckersall SJ, Spreadbury PL. Severe forearm oedema after intravenous infusion with a IMED 960 infusion pump. Eur J Anaesthesiol 1996;13:39-41.

30. Eroglu A, Uzunlar H. Forearm compartment syndrome after intravenous mannitol extravasation in a carbosulfan poisoning patient. J Toxicol Clin Toxicol 2004;42:649-52.

31. Garcia-Alvarez F, Bello ML, Albareda J, Seral F. [Neonatal tumoration in the secondary to calcium extravasation]. Rev Esp Pediatr 1999;55:279-80.

32. Gibboney W, Lemons JA. Necrotizing fasciitis of the scalp in neonates. Am J Perinatol 1986;3:58-60.

33. Govind B, Tete PI, Thomas N. Percutaneous central line extravasation masquerading as an abscess. Indian Pediatr 2014;51:309-10.

34. Grabois FS, Voievdca T, Aqcuavita A, Kizlansky V, Saint Genez D, Vidaurreta S. [Use of sterile petrolatum for extravasation injury in a premature infant]. Arch Argent Pediatr 2008;106:533-5.

35. Handler EG. Superficial compartment syndrome of the foot after infiltration of intravenous fluid. Arch Phys Med Rehabil 1990;71:58-9.

36. Hankin FM, Louis DS. Surgical management of doxorubicin (Adriamycin) extravasation. J Pediatr Orthop 1984;4:96-9.

37. Harb A, Sawyer A, Pandya A. Erythromycin extravasation mimicking necrotising fasciitis in the infant. Eur J Plast Surg 2010;33:159-61.

38. Hasija N, Hazarika A, Sokhal N, Kumar S. Tissue necrosis of hand caused by phenytoin extravasation: an unusual occurrence. Saudi J Anaesth 2014;8:309-10.

39. Hey DM, Koontz SE. Azithromycin extravasation in a pediatric patient. J Pharm Technol 2005;21:83-6.

40. Hironaga M, Fujigaki T, Tanaka S. Cutaneous calcinosis in a neonate following extravasation of calcium gluconate. J Am Acad Dermatol 1982;6:392-5.

41. Hirsch SD, Powers JM, Rhodes JL. Neonatal soft tissue reconstruction using a bioengineered skin substitute. J Craniofac Surg 2016;28:489-91.

42. Hooke MC. Clinical nurse specialist and evidence-based practice: managing anthracycline extravasation. J Pediatr Oncol Nurs 2005;22:261-4.

43. Kameo SY, Silva GM, Sawada NO, Hardman GL. Hyaluronidase post extravasation of intravenous vincristine: use in children with cancer. Journal of Nursing UFPE 2015;9:9239-45.

44. Khan I, Rizvi SSA, Malik I, Weinberger B, Puvabanditsin S, Hegyi T. Extravasation of intravenous fluid in a preterm neonate. Consultant 2014;54:188-90.

45. Kishi C, Amano H, Shimizu A, Nagai Y, Ishikawa O. Cutaneous necrosis induced by extravasation of hydroxyzine. Eur J Dermatol 2014;24:131-2.

46. Kuensting LL. Treatment of intravenous infiltration in a neonate. J Pediatr Health Care 2010;24:184-8.

47. Kumar RJ, Pegg SP, Kimble RM. Management of extravasation injuries. ANZ J Surg 2001;71:285-9.

48. Lee HJ, Kwon SH, Choi JW, Park KC, Youn SW, Huh CH, et al. The management of infantile extravasation injury using maternal platelet-rich plasma. Pediatr Dermatol 2013;30:e114-7.

49. Lehr VT, Lulic-Botica M, Lindblad WJ, Kazzi NJ, Aranda JV. Management of infiltration injury in neonates using duoderm hydroactive gel. Am J Perinatol 2004;21:409-14.

50. Leung PC, Cheng CY. Extensive local necrosis following the intravenous use of X-ray contrast medium in the upper extremity. Br J Radiol 1980;53:361-4.

51. Llinares ME, Bermudez M, Fuster JL, Diaz MS, Gonzalez CM. Toxicity to topical dimethyl sulfoxide in a pediatric patient with anthracycline extravasation. Pediatr Hematol Oncol 2005;22:49-52.

52. Martin PH, Carver N, Petros AJ. Use of liposuction and saline washout for the treatment of extensive subcutaneous extravasation of corrosive drugs. Br J Anaesth 1994;72:702-4.

53. Meszes A, Talosi G, Mader K, Orvos H, Kemeny L, Csoma ZR. Lesions requiring wound management in a central tertiary neonatal intensive care unit. World J Pediatr 2017;13:165-72.

54. Mohr LD, Reyna R, Amaya R. Neonatal case studies using active leptospermum honey. J Wound Ostomy Continence Nurs 2014;41:213-8.

55. Morrison WA, Hurley JV, Ahmad TS, Webster HR. Scar formation after skin injury to the human foetus in utero or the premature neonate. Br J Plast Surg 1999;52:6-11.

56. Mukherjee GD, Guharay BN. Digital gangrene and skin necrosis following extravasation of infusion fluid. J Indian Med Assoc 1977;68:77-9.

57. Nissim L, Gilbertson-Dahdal D. An unusual complication of an infiltrated intravenous catheter: heterotopic ossification in a newborn. J Radiol Case Rep 2008;2:13-5.

58. Onesti MG, Carella S, Maruccia M, Marchese C, Fino P, Scuderi N. A successful combined treatment with dermal substitutes and products of regenerative medicine in a patient affected by extravasation injury from hypertonic solution. In Vivo 2012;26:139-42.

59. O'Reilly C, McKay FM, Duffty P, Lloyd DJ. Glyceryl trinitrate in skin necrosis caused by extravasation of parenteral nutrition. Lancet 1988;2:565-6.

60. Ozcan A, Baratali E, Meral O, Ergul AB, Aslaner H, Coskun R, et al. Bullous dermatitis and skin necrosis developing after adrenalin extravasation. Eurasian J Med 2015;47:226-8.

61. Pantelides NM, Shah AK. Extravasation injury: a simple technique to maintain limb elevation within a Neonatal Intensive Care Unit. J Neonatal Nurs 2013;19:243-5.

62. Park HJ, Kim KH, Lee HJ, Jeong EC, Kim KW, Suh DI. Compartment syndrome due to extravasation of peripheral parenteral nutrition: extravasation injury of parenteral nutrition. Korean J Pediatr 2015;58:454-8.

63. Phillips RA, Andrades P, Grant JH, Ray PD. Deep dopamine extravasation injury: a case report. J Plast Reconstr Aesthet Surg 2009;62:e222-4.

64. Raffaella C, Annapaola C, Tullio I, Angelo R, Giuseppe L, Simone C. Successful treatment of severe iatrogenic calcinosis cutis with intravenous sodium thiosulfate in a child affected by T-acute lymphoblastic leukemia. Pediatr Dermatol 2009;26:311-5.

65. Ravenel SD. Cellulitis from extravasation of calcium gluconate simulating osteomyelitis. Am J Dis Child 1983;137:402-3.

66. Reilly JJ, Neifeld JP, Rosenberg SA. Clinical course and management of accidental adriamycin extravasation. Cancer 1977;40:2053-6.

67. Reynolds BC. Neonatal extravasation injury: case report. Infant 2007;3:230-2.

68. Roberts JR. Cutaneous and subcutaneous complications of calcium infusions. JACEP 1977;6:16-20.

69. Rosales CM, Jackson MA, Zwick D. Malassezia furfur meningitis associated with total parenteral nutrition subdural effusion. Pediatr Dev Pathol 2004;7:86-90.

70. Roth W, Eschertzhuber S, Gardetto A, Keller C. Extravasation of propofol is associated with tissue necrosis in small children. Paediatr Anaesth 2006;16:887-9.

71. Rustogi R, Mill J, Fraser JF, Kimble RM. The use of Acticoat in neonatal burns. Burns 2005;31:878-82.

72. Salameh Y, Shoufani A. Full-thickness skin necrosis after arginine extravasation - a case report and review of literature. J Pediatr Surg 2004;39:e9-11.

73. Samiee-Zafarghandy S, van den Anker JN, Ben Fadel N. Topical nitroglycerin in neonates with tissue injury: a case report and review of the literature. Paediatr Child Health 2014;19:9-12.

74. Sanpera I, Fixsen JA, Hill RA. Injuries to the physis by extravasation. A rare cause of growth plate arrest. J Bone Joint Surg Br 1994;76:278-80.

75. Santoshi JA, Pallapati SC, Thomas BP. Hand contracture: an unusual sequel of intravenous fluid extravasation in the neonatal period. J Postgrad Med 2008;54:244-5.

76. Schafer T, Kukies S, Stokes TH, Levin LS, Donatucci CF, Erdmann D. The prepuce as a donor site for reconstruction of an extravasation injury to the foot in a newborn. Ann Plast Surg 2005;54:664-6.

77. Schie JC, Goodman KL. Treatment of neonatal extravasation injuries using non-contact, low-frequency ultrasound: development of a new treatment protocol. Newborn Infant Nurs Rev 2013;13:42-7.

78. Schumacher HR, Osterman AL, Choi SJ, Weisz PB. Calcinosis at the site of leakage from extravasation of calcium disodium edetate intravenous chelator therapy in a child with lead poisoning. Clin Orthop Relat Res 1987:221-5.

79. Sharief N, Goonasekera C. Soft tissue injury associated with intravenous phenytoin in a neonate. Acta Paediatr 1994;83:1218-9.

80. Shenaq SM, Abbase EH, Friedman JD. Soft-tissue reconstruction following extravasation of chemotherapeutic agents. Surg Oncol Clin N Am 1996;5:825-45.

81. Sindal MD, Nakhwa CP. Metastatic Serratia endophthalmitis associated with extravasation injury in a preterm neonate. Oman J Ophthalmol 2015;8:114-6.

82. Siu SLY, Kwong KL, Poon SST, So KT. The use of hyaluronidase for treatment of extravasations in a premature infant. HK J Paediatr 2007;12:130-2.

83. Siwy BK, Sadove AM. Acute management of dopamine infiltration injury with Regitine. Plast Reconstr Surg 1987;80:610-2.

84. Sokol DK, Dahlmann A, Dunn DW. Hyaluronidase treatment for intravenous phenytoin extravasation. J Child Neurol 1998;13:246-7.

85. Sonohata M, Asami A, Tsunoda K, Hotokebuchi T. Purple glove syndrome associated with intravenous phenytoin administration in a patient with severe mental and motor retardation. J Orthop Sci 2006;11:409-11.

86. Sonohata M, Akiyama T, Fujita I, Asami A, Mawatari M, Hotokebuchi T. Neonate with calcinosis cutis following extravasation of calcium gluconate. J Orthop Sci 2008;13:269-72.

87. Soon SL, Chen S, Warshaw E, Caughman SW. Calcinosis cutis as a complication of parenteral calcium gluconate therapy. J Pediatr 2001;138:778.

88. Spenny ML, Moen KY, Dinulos JG. Acute bullous eruption with compartment syndrome due to intravenous infiltration. Arch Dermatol 2004;140:798-800.

89. Stahl S, Lerner A. Compartment syndrome of the forearm following extravasation of mannitol in an unconscious patient. Acta Neurochir 2000;142:945-6.

90. Subedi A, Bhattarai B, Biswas BK, Khatiwada S. Complex Regional Pain Syndrome (CRPS type-1) in an adolescent following extravasation of dextrose containing fluid-an underdiagnosed case. Korean J Pain 2011;24:112-4.

91. Subhani M, Sridhar S, DeCristofaro JD. Phentolamine use in a neonate for the prevention of dermal necrosis caused by dopamine: a case report. J Perinatol 2001;21:324-6.

92. Talbot SG, Rogers GF. Pediatric compartment syndrome caused by intravenous infiltration. Ann Plast Surg 2011;67:531-3.

93. Tilden SJ, Craft JC, Cano R, Daum RS. Cutaneous necrosis associated with intravenous nafcillin therapy. Am J Dis Child 1980;134:1046-8.

94. Tiras U, Erdeve O, Karabulut AA, Dallar Y, Eksioglu HM. Debridement via collagenase application in two neonates. Pediatr Dermatol 2005;22:472-5.

95. Tobin C. Managing an extravasation wound in a premature infant. Wounds UK 2007;3:90-1.

96. Tuncer S, Aydin A, Erer M. Extravasation of calcium solution leading to calcinosis cutis surrounding the dorsal cutaneous branch of the ulnar nerve. J Hand Surg Br 2006;31:288-9.

97. Vanwijck R, Lengele B. [Liposuction as a help for radiologists. Technical note]. Ann Chir Plast Esthet 1994;39:744-9.

98. von Muhlendahl KE. [Complications due to infusions in infants and young children. Lessons from six cases of litigation of the Norddeutsche Schlichtungsstelle fur Arzthaftpflichtfragen]. Monatsschr Kinderheilkd 2012;160:988-91.

99. Wada A, Fujii T, Takamura K, Yanagida H, Matsuura A, Katayama A. Physeal arrest of the ankle secondary to extravasation in a neonate and its treatment by the Gruca operation: a modern application of an old technique. J Pediatr Orthop B 2003;12:129-32.

100. Wiegand R, Brown J. Hyaluronidase for the management of dextrose extravasation. Am J Emerg Med 2010;28:257.e1-2.

101. Wolfe MS, North ER. Extravasation of injected calcium solution leading to calcifications in the upper extremity of the neonate. Report of a case. J Bone Joint Surg Am 1983;65:558-9.

102. Wong AF, McCulloch LM, Sola A. Treatment of peripheral tissue ischemia with topical nitroglycerin ointment in neonates. J Pediatr 1992;121:980-3.

103. Wong KY, Ching D, Milroy C. Iatrogenic calcinosis cutis following a neonatal extravasation injury. In: 12th Congress of the European Society of Plastic, Reconstructive and Aesthetic Surgery (ESPRAS 2014), 6-11 July 2014*:* 2015; Edinburgh, Scotland; 2015: 33.

104. Yamamoto Y, Igawa H, Minakawa H, Sugihara T, Yoshida T, Minamimoto T, et al. Reconstruction of iatrogenic dorsal skin defects of hands with reverse forearm flaps. Eur J Plast Surg 1994;17:104-5.

105. Yosowitz P, Ekland DA, Shaw RC, Parsons RW. Peripheral intravenous infiltration necrosis. Ann Surg 1975;182:553-6.

106. Zenk KE, Dungy CI, Greene GR. Nafcillin extravasation injury. Use of hyaluronidase as an antidote. Am J Dis Child 1981;135:1113-4.
