# Supplementary material for: Treating extravasation injuries in infants and young children: a scoping review and survey of UK NHS practice
Source: BMC Pediatr. 2019 Jan 7;19:6. doi: 10.1186/s12887-018-1387-1 (PMC6323695; doi:10.1186/s12887-018-1387-1)
Supplement: Supplementary file 4 — Comparative study and non-comparative group study details (DOCX 75 kb) [file 12887_2018_1387_MOESM4_ESM.docx]

Additional file 4 - Comparative study and non-comparative group study details

Table 1 Prospective comparative studies of extravasation treatments

| **Study details** | **Population characteristics** | **Injury details** | **Intervention** | **Results** |
| --- | --- | --- | --- | --- |
| **Authors**  Brown et al 1979 {Brown, 1979 #1874}  **Design**  Quasi-randomised study. Treatment allocation changed with the calendar month. The study ran for 6 months. Each of the three treatments was therefore used for two full months.  **Setting**  Children's Hospital in Pennsylvania, USA  **Sample size**  **34** | **Age**  Range: 5 days to 12 years. 22 were aged <1 year  **Comorbidities**  Varied range, with respiratory distress syndrome, leukaemia, septicaemia, necrotising enterocolitis and Wilm's tumour among the most frequent  **Duration of IV**  NR  **Mean time to treatment**  Not reported | **Method of delivery**  Varied, but mostly infusions  **Types of eligible injury**  Only children with skin loss: partial (e.g. blistering or discolouration) or full thickness  **Sites**  Mostly dorsum of foot or the hand. Some injuries on shin, ankle, wrist, scalp, arm and abdomen.  **Infusates**  Varied, but mostly parenteral nutrition (0.5 to 2% amino acids in 10 to 12.5% glucose solution) sometimes with antibiotics. | All patients had their i.v. therapy stopped, the apparatus removed, and the affected area elevated. Patients were then allocated one of the following treatments:  For patients with partial-thickness skin loss:  1. Apply silver sulfadiazine cream every 8 hours and cover with semi-permeable dressing. Wash area with sterile saline between applications, or  2. Apply povidone-iodine ointment every 8 hours and cover with semi-permeable dressing. Wash area with sterile saline between applications, or  3. Wash area with sterile saline every 8 hours but keep dry and covered with “Sta-Tite” in the intervals.  For patients with full-thickness skin loss:  1. Apply silver sulfadiazine cream every 4 hours. Wash area with sterile saline between applications, cover with “Sta-Tite” in the intervals, or  2. Apply wet-to-dry povidone-iodine solution dressings, covered with “Sta-Tite”, every 4 hours, or  3. Apply wet-to-dry saline dressings, covered with “Sta-Tite”, every 4 hours. | **Time for wound healing**  Silver sulfadiazine: ranged between 3-10 weeks in the 11 children with full thickness loss and 1-5 weeks in the 4 children with partial thickness loss.  Povidone-iodine: ranged between 2-6 weeks in the 6 children with full thickness loss and 3 weeks in the 1 child with partial thickness loss.  Saline: ranged between 4-8 weeks in the 4 children with full thickness loss and 2-3 weeks in the 3 children with partial thickness loss.  “No significant difference in the rate of healing among the 3 treatment protocols is readily apparent. Nevertheless, primary healing occurred in all patients without the need for skin grafting and without loss of function of the affected part”  4 of the full thickness group died. A further 2 patients died before receiving treatment |
| **Authors**  Hanrahan 2013 {Hanrahan, 2013 #279}  **Design**  Before-and-after study of the implementation of a guideline for using hyaluronidase.{Hanrahan, 2012 #7118} Other outcomes included costs, knowledge, extravasation incident reports, hyaluronidase usage reports  **Setting**  Children's Hospital in Iowa, USA  **Sample size**  113 | **Age**  **Not reported (‘paediatric population’)**  **Comorbidities**  **Not reported**  **Duration of IV**  NR  **Mean time to treatment**  Pre implementation: 125 mins (SD=75)  Post implementation: 76 min (SD=38) | Injury details not reported | Hyaluronidase (n=37) or no hyaluronidase (n=76)  Most of the no hyaluronidase group were treated before the guideline was implemented and most of the hyaluronidase group were treated post-implementation. An increase in the frequency of treatment with hyaluronidase was reported from baseline (9%) to implementation (63%). | Agency for Healthcare Research and Quality (AHRQ) Common Format harm scores were used as an outcome measure.  To determine if treatment resulted in less harm, subjects were collapsed into two groups: those receiving hyaluronidase (*n* =37) and those who did not (*n* = 76). Mean harm scores were similar: 5.29 and 5.27, respectively.  No events resulted in permanent harm, severe permanent harm, or deaths (harm score > 6). |

Table 2 Non-comparative studies of conservative management interventions for extravasation injuries

| **Study details** | **Population characteristics** | **Injury details** | **Intervention** | **Results** |
| --- | --- | --- | --- | --- |
| **Authors**  An and Ning 2015{An, 2015 #101}  **Design**  Retrospective case series  **Setting**  Children's hospital emergency department, China  **Sample size**  6 | **Age**  **Mean 4 months ( range 1 to 9 months)**  **Comorbidities**  Respiratory tract infection  **Duration of IV therapy**  2-3 days  **Mean time to treatment**  NR | **Method of delivery**  **NR**  **Types of injury**  Swelling, masses  **Sites**  All scalp  **Infusates**  Mezlocillin and sulbactam sodium | Wet-hot compresses by small sterile gauze 3-4 times per day, with temperature at 40-45 degrees and duration of 20-30 minutes per session. Next, mucopolysaccharide polysulphate (MPS) cream was applied topically folllettowed by tender massage for 3-5 minutes. | After a median duration of 3 days’ treatment, all patients had complete fluid absorption and were discharged without adverse outcomes at 3 months. |
| **Authors**  Moon et al 2012{Moon, 2012 #334}  **Design**  Retrospective case series  **Setting**  Plastic Surgery department, South Korea  **Sample size**  13 (14 injuries) | **Age**  **20 days (31 weeks gest) [14 to 50 days (28 to 35 weeks gest)]**  **Comorbidities**  Prematurity  **Duration of IV therapy**  NR  **Mean time to treatment**  NR | **Method of delivery**  **NR**  **Types of injury**  Full thickness defects  **Sites**  Hand/wrist (5), ankle/foot (8), elbow (1)  **Infusates**  Total parenteral nutrition | Local conservative management was given with healing by secondary intention. An antibiotic and anti-inflammatory ointment mixture was topically applied to the whole region of the wound site, followed by a dressing. This was performed twice a day during the acute phase and once a day during the convalescent phase. Systemic prophylactic antibiotics were also given. The necrotic tissue was removed when it was clearly demarcated. After wound closure, topical oil moisturization and mild compression were applied. | The defects had completely closed 14 to 55 days after injury, the vast majority by wound contraction. Parents were shown photos of the initial defects and the final scar. The degree of the parents’ satisfaction was excellent in 9 cases of pinpoint scars and linear scars, good in 3 cases of depressed or mild contracted linear scars, and fair in 2 cases of round hypertrophic scars. |
| **Authors**  Mu et al 1999{Mu, 1999 #1150}  **Design**  Case series (unclear whether retrospective or prospective)  **Setting**  Paediatric department, Taiwan  **Sample size**  9 | **Age**  **NR (neonates)**  **Comorbidities**  All had neonatal hypocalcaemia. Premature (4), perinatal asphyxia (2)  **Duration of IV therapy**  Range 5-11 days  **Mean time to treatment**  NR | **Method of delivery**  **NR**  **Types of injury**  Calcinosis cutis  **Sites**  Wrist (4), forearm (4), elbow (3), ankle (3)  **Infusates**  Calcium gluconate | Wet dressing and localised massage, followed by rehabilitation programme. | It took 18 to 50 days for functional recovery. 5 lesions still had cosmetic residuals but none required a skin graft. |
| **Authors**  Nandiolo-Anelone et al. 2014 {Nandiolo-Anelone, 2014 #242}  **Design**  **Retrospective case series**  **Setting**  Children's Hospital, Ivory Coast  **Sample size**  15 | **Age**  **Mean 3.6 days. Range 1 to 9 days**  **Comorbidities**  **Maternal and foetal infections (6), foetal distress (4), respiratory distress (4), premature birth (2), denutrition (1), oesophageal atresia (1).**  **Duration of IV**  NR  **Time to injury treatment**  Mean 3.93 days, range 1 to 9 days | **Method of delivery**  **NR**  **Types of injury**  Stage III (6) and IV (9) extravasation injuries  **Sites**  Upper (9) and lower limbs (6)  **Infusates**  Serum 10% glucose and calcium chloride | Alcohol based dressing 48h to 72h (6), pre-surgical pro-inflammatory dressing up to 15 days (6), graft excision (4 primary, 4 secondary), incl. 1 with fasciotomy | Total scores on Vancouver Scar Scale (VSS): 53% with scores 0-1, 26% with scores 2-4, 13% with score 9, 6% lost to follow up.  2 deaths: linked to prematurity (1) and **oesophageal atresia (1).** |
| **Authors**  Sawatzky-Dickson and Bodnaryk 2006{Sawatzky-Dickson, 2006 #814}  **Design**  Prospective case series  **Setting**  Neonatal intensive care unit, Canada  **Sample size**  9 | **Age**  **1 to 40 days (24 to 40 weeks gestation)**  **Comorbidities**  NR  **Duration of IV therapy**  NR  **Mean time to treatment**  NR | **Method of delivery**  **NR**  **Types of injury**  Sloughing or necrosis. Stage 3 or 4 injuries.  **Sites**  Foot (5), hand (3), forearm (1)  **Infusates**  blood transfusion, sodium bicarbonate, parenteral nutrition, dextrose | Wound care protocol developed by the authors. Aqueous gel was applied to coat the area of tissue damage but not the surrounding skin. A hydrofibre sheet was placed over the gel. A thin hydrocolloid dressing covered the area for 7 days (or changed sooner if necessary). Antibiotics were given for wound infections. | Wound healing times ranged from 1 to 6 weeks. No wounds showed any signs of infection. 1 patient died before wound healed.  Notes on research design: “the number of injuries occurring in a year is small so a randomized controlled trial was not possible” |
| **Authors**  Sung & Lee 2016{Sung, 2016 #24}  **Design**  Retrospective case series  **Setting**  Plastic Surgery Department, South Korea  **Sample size**  12 | **Age**  **30 to 39 weeks gestation**  **Comorbidities**  9 were preterm  **Duration of IV therapy**  NR  **Mean time to treatment**  Between 1 and 10 hours for all but 1 patient (52 hours) | **Method of delivery**  **NR**  **Types of injury**  Skin discolouration (7), bleb (2), necrosis (2) swelling (1). 9 eventually progressed to full thickness open wounds  **Sites**  Wrist (5), ankle (5), hand (1), antecubital (1)  **Infusates**  Parenteral nutrition | Multiple wound punctures (using scalpel blade tip) were made and a hydrocolloid dressing applied. This was changed every 6 hours on first day and with decreasing frequency thereafter. Debridement performed gradually when devitalised tissue began to be demarcated and autolysed – done with a scalpel and without anaesthesia. Mean duration of treatment was 25 days. After healing, silicone gel was recommended for 3 months to prevent hypertrophic scars. | Two of the 12 patients presented with necrotic lesions; nine patients eventually progressed to full thickness open wounds.  Wound healing times ranged from 8 to 41 days. There were no secondary infections and minor scarring.  Parents were “satisfied with the final results”.  There was one contracture of the wrist.  Notes on research design: Controlled prospective studies necessary to confirm findings. |
| **Authors**  Wang 2007 {Wang, 2007 #3275}  **Design**  Retrospective case series  **Setting**  NR, USA  **Sample size**  17 | **Age**  **NR**  **Comorbidities**  NR  **Duration of IV therapy**  NR  **Mean time to treatment**  NR | **Method of delivery**  **Injections**  **Types of injury**  Swelling (13), pain (4)  **Sites**  Arm (10), hand (3), shoulder (1), ankle (1), foot (1), groin (1)  **Infusates**  Contrast agent | Cold compresses and elevation. | All wounds healed without necrosis. |

Table 3 Non-comparative studies of flush-out techniques for extravasation injuries

| **Study details** | **Population characteristics** | **Injury details** | **Intervention** | **Results** |
| --- | --- | --- | --- | --- |
| **Authors**  Andres et al 2006{Andres, 2006 #761}  Published in Spanish  **Design**  Retrospective case series  **Setting**  **Paediatric Hospital, Spain**  **Sample size**  **15 (includes 8 patients who received artificial skin, see** Table 5**)** | **Age**  **Mean 3 years**  **Comorbidities**  1 with necrotising enterocolitis, 1 with hyaline membrane disease, 5 oncology patients, 1 severe head trauma and 1 liver transplant patient  **Duration of IV therapy**  NR  **Mean time to treatment**  <24 hours | **Method of delivery**  **NR**  **Types of injury**  Not reported for whole cohort though 5 patients had established necrosis  **Sites**  Dorsum of hand or foot (14), forehead (1)  **Infusates**  Parenteral nutrition (7), calcium gluconate (4), doxorubicin (4) | For cases of less than 24 hours, the Gault method with saline (500cc) was used. In 2 patients hyaluronidase was used before saline flush out.  Includes 8 patients who received artificial skin, see Table 5. | 7 of the 10 patients treated by the Gault method avoided necrosis and recovered fully. |
| **Authors**  Casanova 2001 et al{Casanova, 2001 #1079}  **Design**  Retrospective case series  **Setting**  Plastic Surgery department, France  **Sample size**  14 | **Age**  **20 days (1 day to 6 months)**  **(mean weight 2.5kg)**  **Comorbidities**  **6 babies were premature**  **Duration of IV therapy**  NR  **Mean time to treatment**  3 to 12 hours | **Method of delivery**  **NR**  **Types of injury**  Swelling, discolouration, skin damage, blisters, induration  **Sites**  foot/ankle (9), hand/wrist (3), elbow (1), forehead (1)  **Infusates**  Dopamine (9), Caffeine (2), Beta-blocker (1), calcium (1), Calcium and amikacine (1) | Gault procedure with hyaluronidase under general anaesthesia in 11 cases. Mild aspiration with a 2mm microcannula, following a liposuction technique, was then performed through the micro incisions. Aspiration with a 20 mm syringe or by mild mural aspiration. Procedure repeated several times, rinsing the area with saline after each infiltration of hyaluronidase. Extravasation site protected with an oily dressing. In two cases saline was used instead of hyaluronidase. In one case, only the hyaluronidase flush-out was performed. | “Eleven patients improved, with no skin involvement in ten cases. In one case a pre-existing blister subsided and healed. Three patients developed skin necrosis, which was treated and healed spontaneously.” |
| **Authors**  Ching 2014{Ching, 2014 #2364}; Wong 2015 {Wong, 2015 #5882}  **Design**  Retrospective case series  **Setting**  Plastic Surgery department, UK  **Sample size**  69 | **Age**  **Mean 36 months (1 day to 17 years)**  **Comorbidities**  Prematurity (40%), gastrointestinal (21%), cardio-respiratory (16%), sepsis (16%), other (7%)  **Duration of IV therapy**  NR  **Mean time to treatment**  4 hours (range 0.2 to 24) | **Method of delivery**  **NR**  **Types of injury**  NR  **Sites**  Upper limb in 65% of cases (40% on the dorsum of hand), 25% lower limb and 10% other.  **Infusates**  32% maintenance fluids (glucose, or sodium or potassium chloride), 23% TPN, 45% others | 62% of patients received an early saline washout using the Gault technique. The technique involved creating multiple skin punctures around the periphery of the injury area with an atraumatic cannula and flushing each puncture with 0.9% sodium chloride. All the washouts also used hyaluronidase. | None of the patients who received Gault technique developed complications.  Of the remainder, there were 3 cases of associated infection, 1 case of ischaemic toe with subsequent digit amputation and 1 case of calcinosis cutis (involving prolonged hospitalisation and readmission for secondary infection).  Notes on research design: “The optimal extravasation management is uncertain due to ethical considerations limiting controlled research.”  “A centralised register of extravasation events would be a useful means to monitor, assess, and review outcomes.” |
| **Authors**  Gault 1993{Gault, 1993 #1431}  **Design**  Retrospective case series  **Setting**  Plastic Surgery unit, UK  **Sample size**  96 (includes patients needing surgery see Table 6) | **Age**  **Mean 10 years (range 0 to 70 years).** The study includes some adults but the mean age (and range) suggests most of the population were children.  **Comorbidities**  NR  **Duration of IV therapy**  NR  **Mean time to treatment**  44 patients within 24 hours | **Method of delivery**  **NR**  **Types of injury**  NR  **Sites**  Varied greatly but mostly hand/forearm or foot/ankle.  **Infusates**  Varied greatly but mostly calcium, parenteral nutrition, dextrose, vincristine, daunorubicin or doxorubicin | “Early referral” group: of the 44 patients seen within 24 hours, 37 were treated with saline flush-out (500ml) following prior infiltration with hyaluronidase. 1 with liposuction alone, and 6 with both. Prophylactic antibiotics also used for immunosuppressed. Following flush-out a layer of jelonet and betadine soaked gauze was applied, and limb elevation for 1 day.  “Late referral” group needed extensive reconstructive surgery, see Table 6. | Flush-out group (early referral): no tissue damage (39), minor skin necrosis or delayed healing (5). |
| **Authors**  Ghanem et al 2015{Ghanem, 2015 #150}  **Design**  Prospective case series (audit)  **Setting**  Children's Hospital, UK  **Sample size**  78 (82 injuries) | **Age**  **Mean 3.2 years, median 0.2 years. Range 1 day to 16.7 years**  **Comorbidities**  **NR**  **Duration of IV therapy**  NR  **Time to injury treatment**  **Mean** 8.3 hours | **Method of delivery**  **89% peripheral lines, 9% central lines, 2% other**  **Types of injury**  NR  **Sites**  Varied, but most were the upper limbs (60%) or lower limbs (30%)  **Infusates**  Varied, though TPN for 46% of the extravasation injury group (n=48). Antibiotics and sodium chloride caused over half the injuries classed as infiltration. No chemotherapeutic extravasation injuries. | Evaluation of a hospital guideline of early referral to plastic surgeons and washout of high risk cases.  Extravasation injuries were diagnosed in 48/82 cases (i.e. vesicant involved). The rest were classed as infiltration injuries. 22 of the 48 extravasation injuries required washout with hyaluronidase, the remainder were treated conservatively with elevation and analgesia.  None of the infiltration injuries required washout. | Limited outcome data reported. Three patients had tissue necrosis. There was satisfactory healing with no requirement for surgical intervention. 2 of these 3 cases were referred later than 24 hours after the injury. |
| **Authors**  Harris et al 2001{Harris, 2001 #1084;Harris, 2002 #1018}  **Design**  Prospective case series (reported in a letter)  **Setting**  Neonatal unit, UK  **Sample size**  56 confirmed injuries from 82 referrals | **Age**  **NR (neonates)**  **Comorbidities**  NR  **Duration of IV therapy**  NR  **Time to injury treatment**  Unclear “immediately assessed” | **Method of delivery**  **NR**  **Types of injury**  **NR**  **Sites**  **NR**  **Infusates**  Parenteral nutrition, inotropes, dextrose, calcium, potassium, and bicarbonate | Modification of Gault's saline flush out technique: 500 ml saline, to exit via puncture wounds | No episodes of skin or soft tissue loss were recorded and no reconstructive surgery was required. |
| **Authors**  Kostogloudis et al 2015{Kostogloudis, 2015 #119}  **Design**  Case series (unclear whether or not prospective)  **Setting**  Neonatal intensive care unit, Greece  **Sample size**  34 | **Age**  **Mean age 11.6 days. 4 neonates were extremely preterm, 9 were very preterm, 14 were late preterm, 7 were full-term. Gestational age range range: 24-42 weeks**  **Comorbidities**  NR  **Duration of IV therapy**  NR  **Time to injury treatment**  Range 10-30 minutes | **Method of delivery**  **Peripheral IV infusion**  **Types of injury**  Neonates with stage III and IV extravasation injuries were included in the study  **Sites**  Ankle (22 patients), dorsum of hand (6), dorsum of foot(3) and thigh (3)  **Infusates**  Parenteral nutrition (28), dextrose 10% (4), Cephalosporin (2) | Normal saline flush out (mean 60ml, range 10 to 160 ml), 2 to 6 full thickness incisions made. Dressing (paraffin and povidone iodine soaked gauze) and elevation for 24 hours. Dressing changed daily until healing complete. | Wound healing in 1 to 25 days. All infants responded well to washout - clinical findings subsided significantly within 24 hours. 21 neonates showed no signs of soft tissue damage 24 hours after treatment and only minor findings, such as blistering and epidermolysis were still present in seven neonates in the next few days.  Ischaemic signs recorded in six neonates by day 2, but gradually subsided within 25 days. Incisions healed uneventfully within 7 to 13 days and with minimal scar formation.  Hypoplasia of the toenails noted in one case at 26 months. One neonate had compartment syndrome - emergency fasciotomies, followed by saline irrigation were performed. All incisions healed uneventfully by secondary intention, resulting in fully functional upper extremities with aesthetically acceptable scar formation. |

Table 4 Non-comparative studies of other (non flush-out) hyaluronidase interventions for extravasation injuries

| **Study details** | **Population characteristics** | **Injury details** | **Intervention** | **Results** |
| --- | --- | --- | --- | --- |
| **Authors**  Cochran et al 2002  {Cochran, 2002 #1020}  **Design**  Retrospective database study  **Setting**  Radiology department, USA  **Sample size**  8 children (study also reported data for adults) | **Age**  **3 months to 9 years**  **Comorbidities**  NR  **Duration of IV therapy**  NR  **Mean time to treatment**  NR | **Method of delivery**  **4 were manual injection**  **Types of injury**  NR  **Sites**  NR  **Infusates**  All contrast material | 2 were treated with hyaluronidase | No results data relating to all 8 children were presented other than “one extravasation had a prolonged course”; brief details were reported for this child. |
| **Authors**  Yan et al 2014{Yan, 2014 #170}  **Design**  Retrospective case series  **Setting**  Neonatal department, China  **Sample size**  13 | **Age**  **Mean 26 days (range 5 to 150)**  **Comorbidities**  Prematurity (5), pneumonia (4), gastrointestinal disorders (2), malnutrition (1), neonatal jaundice(1)  **Duration of IV therapy**  NR  **Mean time to treatment**  Median 6.4 hours for 12 cases. For the calcium chloride patient the hyaluronidase was given after 14 hours. | **Method of delivery**  **NR**  **Types of injury**  Swelling in all 13, with erythema (5), blister (3), necrosis (1).  **Sites**  Hand (4), leg (3), forearm (3) wrist, armpit, scalp (all 1)  **Infusates**  Total parenteral nutrition (9), calcium chloride, 10% dextrose, immunoglobin , para-aminomethylbenzoic acid +etamylate (all 1) | Hyaluronidase injections and hirudoid cream. A 25-gauge needle was used and a total of 1-ml solution (150 U/ml) of hyaluronidase was divided into 5 0.2-ml injections: one in the centre while 4 along the edge of the extravasation sites. Some cases needed another injection several hours after the first injection (contained five 0.2cc injections). Hirudoid cream was massaged around the affected area. | “After the treatment, the symptoms improved, and no complications were noted at the follow-up within 48 hours and 3 months. Negligible loss of functional movements of the fingers, hands, arms, feet, or legs was noticed.”  For the calcium chloride patient a scar developed 2 days after treatment, and calcinosis developed 3 weeks after hospital discharge.  3 cases were lost to follow up.  No adverse drug effects were reported. |
| **Authors**  Crowther et al 2011{Crowther, 2011 #408}  **Design**  **Retrospective hospital database study**  **Setting**  Children's Hospital, USA  **Sample size**  90 | **Age**  **Mean 28 days (range 2 to 93) for the 38 patients with extravasation of parenteral nutrition**  **Comorbidities**  NR  **Duration of IV therapy**  NR  **Time to injury treatment**  NR | **Method of delivery**  **Mostly peripheral IV lines**  **Infusates**  42% parenteral nutrition, other agents such as dopamine, dextrose, potassium chloride, contrast media, ciprofloxacin and fentanyl each made up ≤5% of total  **Types of injury**  NR  **Sites**  NR | “Treated with hyaluronidase” for 31 of the 38 parenteral nutrition extravasations. No further details were reported about how hyaluronidase was used.    Other treatments included elevation, hot or cold compresses, and analgesic use but these were not consistently documented. | 46% full recovery, remainder were referred to wound care (10%) or did not have a documented outcome (44%) |

Table 5 Non-comparative studies of artificial skin interventions for extravasation injuries

| **Study details** | **Population characteristics** | **Injury details** | **Intervention** | **Results** |
| --- | --- | --- | --- | --- |
| **Authors**  Andres et al 2006{Andres, 2006 #761}  Published in Spanish  **Design**  Retrospective case series  **Setting**  **Paediatric Hospital, Spain**  **Sample size**  **15 (includes 10 flush-out patients, see** Table 3**)** | **Age**  **Mean 3 years**  **Comorbidities**  1 with necrotising enterocolitis, 1 with hyaline membrane disease, 5 oncology patients, 1 severe head trauma and 1 liver transplant patient  **Duration of IV therapy**  NR  **Mean time to treatment**  <24 hours | **Method of delivery**  **NR**  **Types of injury**  Not reported for whole cohort though 5 patients had established necrosis  **Sites**  Dorsum of hand or foot (14), forehead (1)  **Infusates**  Parenteral nutrition (7), calcium gluconate (4), doxorubicin (4) | For cases of less than 24 hours, the Gault method with saline (500cc) was used.  In 5 patients, where necrosis was already established (and 3 where it started after the flush out), the area was covered with artificial skin consisting of 2 membranes: one comprised a three-dimensional porous fibrillar mesh of bovine tendon collagen next to chondroitin-6-sulfate. The other was a thin sheet of silicone. Necrotic tissue was debrided and after 2 or 3 weeks a partial skin graft was performed. | In total, 8 patients needed the artificial skin and skin grafts. All recovered full functionality with only minor scars; no secondary surgery or amputation was necessary. |
| **Authors**  Onesti et al 2012{Onesti, 2012 #388}  **Design**  Case series (reported in a letter). Unclear whether or not prospective.  **Setting**  Plastic surgery department, Italy  **Sample size**  26 | **Age**  **Mean gestational age 32 weeks (range 28-36 weeks)**  **Comorbidities**  Pre-term neonates  **Duration of IV therapy**  Mean 3 days, range 2 days to 1 week  **Time to injury treatment**  NR | **Method of delivery**  **NR**  **Types of injury**  Patients with partial and full-thickness wounds. Cutaneous eschar (8 patients), ulcers, blisters and erythematous wound margins (18)  **Sites**  Dorsum of hand (11), dorsum of foot (6), forearm (3), ankle (1), leg (3), scalp (2)  **Infusates**  Hypertonic solution | Hyalomatrix PA (a dermal substitute composed of 2 layers): first, topical collagenase was applied, then 72 hours later debridement, followed by application of Hyalomatrix PA. After 1 week the area was cleaned and Hyalomatrix PA applied and kept in place for 7 to 11 days. This procedure was continued for a total of 21 days. | 18 patients had restoration of dermal quote and a rapid re-epithelialization process after 21 days.  Patients were followed up for up to 14 months. 4 had pathologic scars, 4 had debilitating scar contractures needing secondary surgery. There were no wound infections. |

Table 6 Non-comparative studies of debridement and plastic surgery interventions for extravasation injuries

| **Study details** | **Population characteristics** | **Injury details** | **Intervention** | **Results** |
| --- | --- | --- | --- | --- |
| **Authors**  Falcone et al 1989  {Falcone, 1989 #1604}  **Design**  Retrospective case series  **Setting**  Plastic Surgery department, USA  **Sample size**  15 (16 injuries) | **Age**  **Mean gestational age 28.5 weeks (range 26 to 33 weeks)**  **Comorbidities**  Preterm, hyaline membrane disease, rule out sepsis, patent ductus arteriosus, necrotising enterocolitis, seizures bronchopulmunary dysplasia  **Duration of IV therapy**  NR  **Mean time to treatment**  NR | **Method of delivery**  **NR**  **Types of injury**  Full thickness injury  **Sites**  Foot (13), hand (2) and scalp (1)  **Infusates**  Parenteral nutrition (9), Electrolyte solution (3), unknown (3) | Enzymatic debridement.  Debridement and topical fibrinolysin/ deoxyribonuclease ointment every 8 hours. After 5-7 days process is repeated until the wound begins to re-epithelialise (3-4 weeks). | All wounds healed completely with no infections and no functional scar contractions at up to 16 months’ follow up. No skin grafts were needed. |
| **Authors**  Gault 1993{Gault, 1993 #1431}  **Design**  Retrospective case series  **Setting**  Plastic Surgery unit, UK  **Sample size**  96 (includes patients receiving flush-out, see Table 3) | **Age**  **Mean 10 years (range 0 to 70 years).** The study includes some adults but the mean age (and range) suggests most of the population were children.  **Comorbidities**  NR  **Duration of IV therapy**  NR  **Mean time to treatment**  44 patients within 24 hours | **Method of delivery**  **NR**  **Types of injury**  NR  **Sites**  Varied greatly but mostly hand/forearm or foot/ankle.  **Infusates**  Varied greatly but mostly calcium, parenteral nutrition, dextrose, vincristine, daunorubicin or doxorubicin | “Early referral” group: 44 patients, see Table 3.  “Late referral” group: 15 of the 51 patients needed extensive reconstructive surgery. | Late referral: no tissue damage (8 patients), minor skin necrosis or delayed healing (17), scar revision (5), skin graft (6), contractures (6), flap coverage required (6), amputation (3 neonates), infection (1). |
| **Authors**  Linder et al 1983{Linder, 1983 #1783;Linder, 1985 #1724}  **Design**  Retrospective case series  **Setting**  Plastic surgery department, USA  **Sample size**  18 (study also included 22 adults) | **Age**  **6 months to 14 years**  **Comorbidities**  Mostly acute myelogenous leukaemia or lymphoma  **Duration of IV therapy**  NR  **Mean time to treatment**  Range 6 to 62 days | **Method of delivery**  **Some IV. drip, some push.**  **Types of injury**  Only patients with extensive injuries, defined as 300 square cm of tissue loss  **Sites**  Upper arm, forearm or hand, foot and leg  **Infusates**  Doxorubicin hydrochloride | Surgery - debridement and wound closure: mostly split-thickness skin grafts or delayed primary closure.  Sodium hypochlorite dressings.  All patients had at least two operations. | The mean time for wound closure was 49 days (10 to 85 days). 3 patients died before wound closure. At least 1 patient needed a split thickness skin graft but could be more as results not given separately for children and adults. One child developed sympathetic dystrophy syndrome. Some children developed permanent joint stiffness. Other negative outcomes are mentioned but unclear if they were experienced by the child sample. |
| **Authors**  Sivrioglu 2014{Sivrioglu, 2014 #226}  **Design**  Retrospective case series  **Setting**  Plastic Surgery Department, Turkey  **Sample size**  9 | **Age**  **26 days (range 1 day to 3 months)**  **Comorbidities**  NR  **Duration of IV therapy**  NR  **Mean time to treatment**  All within 12 hours | **Method of delivery**  **NR**  **Types of injury**  Skin necrosis  **Sites**  Hand/wrist (5), foot (3) and scalp (1)  **Infusates**  Calcium gluconate | Injuries were initially flushed with saline and a cold compress applied. Oily dressings were applied and skin necrosis developed within a week. Debridement with the Versajet^TM^ Hydro surgery system (a waterjet debriding tool) under general anaesthesia. Oily dressings used after debridement. | Wounds healed spontaneously by re- epithelialization. The mean time of full epithelialization was 14 days. At 1 year, minimal scar formation was noted with no hypertrophic scars. |
| **Authors**  Upton et al 1979{Upton, 1979 #1867}  **Design**  Retrospective case series  **Setting**  Various departments in 5 USA hospitals  **Sample size**  7 (study also reported on 24 adults) | **Age**  **5.6 years (1 week to 11 years)**  **Comorbidities**  Varied, including acute leukaemia, head trauma, gastroenteritis  **Duration of IV therapy**  NR  **Mean time to treatment**  1 to 42 days | **Method of delivery**  **Varied**  **Types of injury**  Major injuries with full thickness tissue loss  **Sites**  Hand, scalp, forearm, wrist, foot or ankle  **Infusates**  Varied including dextrose, potassium chloride, doxorubicin hydrochloride, tetracycline | Debridement and skin grafts. All patients needed 2 or more operations. | Below elbow amputation (1), contractures (2), extensor loss (2), hair loss (1), loss of motion (1), reconstruction needed (1) |
| **Authors**  Von Heimburg and Pallua 1998{von Heimburg, 1998 #1192}  Published in German  **Design**  Actual design is retrospective comparative study. But extractable data for 5 infants  **Setting**  Germany, Specialised clinic for plastic-, hand- and burns surgery  **Sample size**  **Extractable: 5 infants (1 case report)** | **Age**  **5 infants (no further details other than minimum age 2 weeks)**  **Comorbidities**  NR  **Duration of IV therapy**  NR  **Mean time to treatment**  19 days (range 2 to 10 weeks) but these figures include 19 adult cases | **Method of delivery**  **NR**  **Types of injury**  Late phase, requiring surgical intervention  **Sites**  Bridge of foot  **Infusates**  NR | 1. Debridement 2. Temporary wound coverage by allogeneic donor-tissue grafts 3. Finally, autologous split-skin graft | After 15 days there was full healing (defined as time at which no further wound-coverage changes were required) in all 5 infants. |
| **Authors**  Weiss 1975{Weiss, 1975 #4350}  **Design**  Retrospective case series  **Setting**  Dept. of premature infants, Israel  **Sample size**  4 | **Age**  **NR (but premature neonates)**  **Comorbidities**  Neonatal hypocalcaemia  **Duration of IV therapy**  Up to 15 days  **Mean time to treatment**  NR, but lesions developed after needle removal | **Method of delivery**  **NR**  **Types of injury**  Localised skin necrosis  **Sites**  All scalp  **Infusates**  Calcium gluconate | Wet dressings and repeated economical debridement | Wounds healed well in 15 to 40 days. Scars were visible but without discolouration. |
