# Supplementary material for: Treating extravasation injuries in infants and young children: a scoping review and survey of UK NHS practice
Source: BMC Pediatr. 2019 Jan 7;19:6. doi: 10.1186/s12887-018-1387-1 (PMC6323695; doi:10.1186/s12887-018-1387-1)
Supplement: Supplementary file 5 — Survey result details (DOCX 43 kb) [file 12887_2018_1387_MOESM5_ESM.docx]

Table 8 Positions of survey responders

| **Position** | **Number (%) of responders** |
| --- | --- |
| Consultant Neonatologist | 31 (48) |
| Nursing staff | 10 (16) |
| Consultant Paediatrician | 8 (13) |
| PICU Consultant | 4 (6) |
| Consultant Paediatric Oncologist | 2 (3) |
| Clinical Nurse Educator | 2 (3) |
| Specialist Registrar | 2 (3) |
| Associate Specialist | 1 (2) |
| Paediatric Registrar | 1 (2) |
| Ward Manager | 1 (2) |
| Neonatal Midwife | 1 (2) |

Figure 2 Percentage of neonatal unit extravasation injuries caused by parenteral nutrition in units indicating parenteral nutrition as the main cause of injuries

Table 9 Extravasation injury treatments used in neonatal units

| **Treatment** | **Number of responses (% of total)** | | | | | **Total number of responders** |
| --- | --- | --- | --- | --- | --- | --- |
|  | **Always** | **Usually** | **Sometimes** | **Rarely** | **Never** |  |
| Elevation of affected area | 12 (29) | 12 (29) | 13 (31) | 4 (10) | 1 (2) | 42 |
| Warm compress | 1(3) | 2 (6) | 3 (8) | 13 (36) | 17 (47) | 36 |
| Cold compress | 1(3) | 0 | 3 (9) | 11 (33) | 18 (55) | 33 |
| Analgesia | 9 (21) | 19 (45) | 10 (24) | 4 (10) | 0 | 42 |
| A specific topical cream or ointment | 0 | 3 (8) | 7 (19) | 6 (17) | 20 (56) | 36 |
| Occlusive dressing | 3 (8) | 6 (15) | 8 (2) | 10 (26) | 12 (31) | 39 |
| Saline irrigation without hyaluronidase | 0 | 6 (15) | 15 (38) | 5 (13) | 13 (33) | 39 |
| Saline irrigation with hyaluronidase | 2 (5) | 9 (23) | 11 (28) | 7 (18) | 11 (28) | 40 |
| Antidotes to specific infusates | 0 | 2 (6) | 2 (6) | 7 (20) | 24 (69) | 35 |

Table 10 Extravasation injury treatments used in principal oncology/haematology units

| **Treatment** | **Number of responses (% of total)** | | | | | **Number of responders** |
| --- | --- | --- | --- | --- | --- | --- |
|  | **Always** | **Usually** | **Sometimes** | **Rarely** | **Never** |  |
| Elevation of affected area | 4 (40) | 3 (30) | 2 (20) | 1 (10) | 0 | 10 |
| Warm compress | 2 (22) | 1 (11) | 5 (56) | 0 | 1 (11) | 9 |
| Cold compress | 3 (33) | 0 | 5 (56) | 0 | 1 (11) | 9 |
| Analgesia | 6 (67) | 1 (11) | 2 (22) | 0 | 0 | 9 |
| A specific topical cream or ointment | 3 (33) | 1 (11) | 3 (33) | 0 | 2 (22) | 9 |
| Occlusive dressing | 0 | 0 | 3 (38) | 1 (13) | 4 (50) | 8 |
| Saline irrigation without hyaluronidase | 1 (11) | 0 | 2 (22) | 2 (22) | 4 (44) | 9 |
| Saline irrigation with hyaluronidase | 2 (22) | 1 (11) | 3 (33) | 1 (11) | 2 (22) | 9 |
| Antidotes to specific infusates | 3 (50) | 0 | 1 (17) | 0 | 2 (33) | 6 |

Table 11 Extravasation injury treatments used in PICUs

| **Treatment** | **Number of responses (% of total)** | | | | | **Number of responders** |
| --- | --- | --- | --- | --- | --- | --- |
|  | **Always** | **Usually** | **Sometimes** | **Rarely** | **Never** |  |
| Elevation of affected area | 4 (80) | 1 (20) | 0 | 0 | 0 | 5 |
| Warm compress | 0 | 0 | 0 | 1 (25) | 3 (75) | 4 |
| Cold compress | 0 | 0 | 0 | 2 (50) | 2 (50) | 4 |
| Analgesia | 3 (60) | 1 (20) | 1 (20) | 0 | 0 | 5 |
| A specific topical cream or ointment | 1 (25) | 0 | 0 | 2 (50) | 1 (25) | 4 |
| Occlusive dressing | 0 | 0 | 0 | 2 (40) | 3 (60) | 5 |
| Saline irrigation without hyaluronidase | 1 (20) | 0 | 3 (60) | 0 | 1 (20) | 5 |
| Saline irrigation with hyaluronidase | 2 (40) | 0 | 2 (40) | 1 (20) | 0 | 5 |
| Antidotes to specific infusates | 0 | 0 | 1 (33) | 0 | 2 (67) | 3 |
